# Advances in Nano‐Immunomodulatory Systems for the Treatment of Acute Kidney Injury

**DOI:** 10.1002/advs.202409190

**Published:** 2025-03-27

**Authors:** Chenli Zhang, Zeli Xiang, Pengfei Yang, Ling Zhang, Jun Deng, Xiaohui Liao

**Affiliations:** ^1^ Department of Nephrology The Second Affiliated Hospital Chongqing Medical University Chongqing 400016 China; ^2^ Department of nephrology Second People's Hospital of Yibin Yibin 644000 China; ^3^ Institute of Burn Research, Southwest Hospital State Key Lab of Trauma and Chemical Poisoning Army Medical University (Third Military Medical University) Chongqing 400038 China

**Keywords:** acute kidney injury, immune microenvironment, immunomodulatory nanomaterials

## Abstract

Acute kidney injury (AKI) occurs when there is an imbalance in the immune microenvironment, leading to ongoing and excessive inflammation. Numerous immunomodulatory therapies have been suggested for the treatment of AKI, the current immunomodulatory treatment delivery systems are suboptimal and lack efficiency. Given the lack of effective treatment, AKI can result in multi‐organ dysfunction and even death, imposing a significant healthcare burden on both the family and society. This underscores the necessity for innovative treatment delivery systems, such as nanomaterials, to better control pathological inflammation, and ultimately enhance AKI treatment outcomes. Despite the modification of numerous immunomodulatory nanomaterials to target the AKI immune microenvironment with promising therapeutic results, the literature concerning their intersection is scarce. In this article, the pathophysiological processes of AKI are outlined, focusing on the immune microenvironment, discuss significant advances in the comprehension of AKI recovery, and describe the multifunctionality and suitability of nanomaterial‐based immunomodulatory treatments in managing AKI. The main obstacles and potential opportunities in the swiftly advancing research field are also clarified.

## Graphical abstract

Based on the biodegradability and the immunological reactiveness, IMNs can be broadly classified into four categories. The first category includes types of biologically derived materials that cause immunomodulatory action and biodegradability like nanoparticles of cell membranes, extracellular vesicles, and exosomes. The second category includes IMNs developed using nanomaterials having known immunoactivity and nonbiodegradable such as metallic nanoparticles and acid‐treated and carboxyl functionalized‐carbon nanotubes (f‐CNTs). The third category of IMNs includes biodegradable and immunologically inert materials like nanoparticles of polymers, lipids, and dendrimers. Lastly, the fourth category of IMNs involves non‐biodegradable and immunologically inert materials involves non‐functionalized CNTs, Atv/PTP‐T Ceria NPs, and Se@BSA NPs.

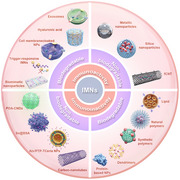

## 1 Introduction

Acute kidney injury (AKI) is a serious kidney disorder marked by a swift reduction in renal function, which is often accompanied by metabolic acidosis, hyperkalemia, volume overload, and/or uremic symptoms caused by a decreased glomerular filtration rate (GFR).^[^ ^1^ ^]^ AKI has high incidence and mortality rates (≈50%), and high medical costs, even with expensive comprehensive supportive treatment.^[^ ^2^ ^]^ Furthermore, the occurrence of severe or recurring AKI frequently results in abnormal renal regeneration and renal fibrosis, hastening the deterioration of kidney function and ultimately leading to the progression of chronic kidney disease (CKD). Common causes of AKI include systemic/local ischemic injury to the kidneys after surgery, sepsis, trauma, and drug toxicity reactions. Dehydration, infections, and toxins linked to acute diseases may also lead to AKI. The pathophysiological mechanisms related to acute kidney damage include inflammatory cell infiltration, excessive production of inflammatory cytokines, oxidative stress, mitochondrial dysfunction, and immune dysregulation.^[^ ^3^ ^]^ Because of the complex pathogenesis of AKI, traditional treatment strategies primarily focus on symptomatic supportive care. However, these therapeutics lack specificity and precision. Therefore, there is a pressing need to develop effective kidney‐targeted AKI treatment methods.

Recent findings suggest that both innate and adaptive immune responses play an important role in the damage and healing of renal tubular cells in AKI. The importance of the abnormal immune microenvironment is increasingly being emphasized in AKI treatment research. Renal injury involves monocytes/macrophages, dendritic cells (DCs), neutrophils, B lymphocytes, as well as T lymphocytes, and the renal cell population of immune cells varies according to the severity of the injury. Moreover, M2 macrophages and regulatory T cells play roles in inhibiting inflammation and tissue repair after kidney damage.^[^ ^4^, ^5^ ^]^ Excessive infiltration of immune cells, excessive production and secretion of inflammatory cytokines, impaired blood flow, and heightened oxidative stress collectively extend inflammation and hinder the integrated repair phase. Consequently, to expedite the repair process in AKI, immunomodulatory interventions are essential to disrupt this deleterious positive feedback loop.

Within this framework, the innovative concept of immunomodulatory nanosystems (IMNs) presents fresh opportunities for the effective and secure treatment of AKI. Previous studies have primarily explored four types of IMNs, with the first being drug delivery nanocarriers, which act as carriers for the controlled transport of immunomodulatory substances to hyperactive immune cells (HICs). For instance, polycations, a synthetic chitosan nanomaterial, can act as efficient gene carriers. The positive charge on polycations can form complexes with gene medications such as siRNA through electrostatic interaction, facilitating the endocytosis of the nanocarrier by effector cells.^[^ ^6^ ^]^ Moreover, various materials, including metal nanoparticles and biologically derived materials such as bilirubin and hyaluronic acid, possess inherent immunomodulatory effects. Metal nanomaterials such as gold and platinum have demonstrated strong immunomodulatory effects, and consequently, have been utilized in the form of IMNs. Ultra‐small nanoparticles of precious metals like gold^[^ ^7^ ^]^ and platinum^[^ ^8^ ^]^ have been created to treat AKI because they possess multiple antioxidase properties. These nanoparticles frequently passively target kidney tissue, aiming to eliminate a variety of reactive oxygen species (ROS). Platinum nanoparticles, in particular, have garnered significant interest because of their broad‐spectrum and highly efficient ROS scavenging activity, mimicking enzymes such as catalase, superoxide dismutase, and NADH coenzyme Q reductase.^[^ ^8^ ^]^ Finally, mixed IMNs comprise a combination of materials from the first three categories, with examples including stimulus‐responsive IMNs, cell membrane‐wrapped nanoparticles (NPs), and biomimetic nanoparticles. Coenzyme Q10 (CoQ10) is a vital electron carrier within the mitochondrial electron transport chain and an efficient ROS scavenger.^[^ ^9^ ^]^ Recently, Liu et al.^[^ ^10^ ^]^ introduced a new formulation, Coenzyme Q10 wrapped in neutrophil cell membranes, known as N‐NPCoQ10, for the treatment of AKI. N‐NPCoQ10 inherits the antigenicity of the neutrophil cell membrane (acting as a nano‐decoy) to capture and counteract complex pathological molecules. During AKI, neutrophil cell membrane signaling molecules respond to inflammatory cues in the kidneys to enable more accurate and targeted administration of CoQ10. Nanoscale IMNs enable them to effectively bind to receptors on cell surface and subsequently be internalized by immune cells, leading to significant therapeutic outcomes. Nevertheless, thorough evaluations of IMNs for the management of AKI are currently lacking, particularly those that specifically examine the use of nanosystem‐based drug delivery and its possible mechanisms of function in AKI treatment.

Herein, we describe the pathophysiological process of AKI, particularly the immune microenvironment, and summarize the strategies based on nanosystems aimed to modify the immune microenvironment. We present valuable perspectives for designing more efficient IMNs for AKI, and explore the current challenges and future prospects related to IMNs for AKI therapy, thereby facilitating their transition into practical application in a clinical setting.

## 2 Pathophysiological Process of AKI

### 2.1 Pathogenic Characteristics of AKI

The primary characteristic of AKI is a sudden increase in serum creatinine (SCr), a decrease in urine output, and impaired kidney function within a few hours to days. The etiology of AKI includes various factors, such as ischemia, sepsis, and nephrotoxicity. Therefore, it is imperative to thoroughly investigate the development and pathological features of AKI while also examining efficient therapeutic strategies. The pathophysiological causes of AKI are intricate and varied, resulting from both systematic and local variables, and are not entirely understood. The underlying processes are complex and thought to be mainly caused by immune dysregulation, excessive inflammatory responses, and the accumulation of ROS leading to tissue damage, cell death, and tissue fibrosis. These pathogenic mechanisms may co‐exist in patients with AKI, thereby complicating the diagnosis and treatment of AKI.

#### 2.1.1 Immune Dysregulation and Excessive Inflammatory Responses

Immune dysregulation and excessive inflammatory responses are significant pathogenic causal factors in the progression of AKI. Under physiological conditions, some innate immune cells, including lymphocytes, macrophages, and DCs, exist in the normal human kidney.^[^ ^11^ ^]^ The immune cells already present in the kidney become active and attract immune cells from the bloodstream to enter the kidney, after the tubular epithelial cells (TECs) and endothelial cells are damaged.^[^ ^12^, ^13^, ^14^ ^]^ The development of AKI depends on the duration and severity of kidney damage.

The initial phase of AKI is tubular cell death, which is caused by various factors such as toxic damage, sepsis, oxidative stress, and ischemia.^[^ ^15^ ^]^Whether organ damage is caused by pathogen invasion during sepsis or aseptic injury, the innate immune system is first activated. Inflammasomes are sensors of the innate immune system and are activated under the stimulation of danger signals. They are composed of a variety of multi‐protein complexes, including Toll‐like receptors (TLRs) and nucleotide‐binding oligomerization domain (NOD)‐like receptors (NLRs), and can trigger inflammation in various diseases, especially immune diseases.^[^ ^16^ ^]^ The basic structure of inflammasomes consists of receptors, adaptors, and effectors. Receptors are classified according to their structural characteristics, including TLRs, NLRs, Absent in Melanoma 2‐Like receptors (ALRs), and Pyrin.^[^ ^17^ ^]^ The adaptor molecule, apoptosis‐associated speck‐like protein (ASC), can recruit and activate caspase‐1, which is an effector.^[^ ^18^ ^]^ The effector molecule pro‐caspase‐1 induces the maturation of pro‐IL‐1β and pro‐IL‐18, leading to pyroptosis.^[^ ^16^ ^]^

Innate immunity primarily identifies inflammasomes or danger signals through pattern recognition receptors (PRRs). In the context of innate immune recognition, there exist two kinds of PRRs: one is responsible for recognizing pathogen‐associated molecular patterns (PAMPs), while the other is for recognizing danger‐associated molecular patterns (DAMPs).^[^ ^19^ ^]^ PAMPs are predominantly conserved molecules located on the exterior of microorganisms, representing structural motifs identified in viruses, fungi, and bacteria, including lipopolysaccharide, flagellin, components of bacterial cell walls, and viral/bacterial nucleic acids.^[^ ^20^ ^]^ By contrast, DAMPs are largely derived from host cells and are capable of transmitting signals related to injury or cell death.^[^ ^21^ ^]^ Kidney‐specific DAMPs encompass Tamm‐Horsfall glycoprotein, crystals, and uromodulin, which can be released as a result of, for instance, tubular injury. Non‐kidney‐specific DAMPs incorporate intracellular particles like the nucleus [comprising histones, high‐mobility group box 1 protein (HMGB1)] and components of the cytosol. Multiple DAMPs are implicated in septic AKI, namely HMGB1, histones, biglycan, decorin, extracellular DNA, and Tamm‐Horsfall glycoprotein.^[^ ^22^ ^]^ In the context of ischemic AKI, DAMPs are primarily released from necrotic cells, including HMGB1, adenosine triphosphate (ATP), DNA, and ribonucleic acid (RNA).^[^ ^23^ ^]^ In the presence of danger signals, PAMPs or DAMPs activate the PRRs located on NLRs or TLRs, consequently triggering the ASC adaptor to activate the effector molecules caspase‐1 or caspase‐11. The activation of these molecules can result in the secretion of pro‐inflammatory cytokines, namely pro‐IL‐1β and pro‐IL‐18,^[^ ^24^, ^25^, ^26^ ^]^ in turn, driving the infiltration of a large number of immune cells into the kidney tissues.^[^ ^27^, ^28^, ^29^ ^]^ For example, in the early stages of AKI, damaged TECs can promote the differentiation of M1 macrophages through the release of exosomal miRNAs. The synthesis of inflammatory factors, including interleukin‐1 beta (IL‐1β), tumor necrosis factor‐alpha (TNFα), and C‐C motif chemokine ligand 2 (CCL2), stimulates the entry of macrophages and T lymphocytes into the kidney interstitium.^[^ ^30^ ^]^ Dying endothelial and tubular epithelial cells emit DAMPs including histones and heat shock proteins, which stimulate the recruitment of neutrophils to the kidneys. Activated platelets and infiltrated neutrophils engage in interactions that lead to the formation of neutrophil extracellular traps (NETs), intensifying tissue damage.^[^ ^31^ ^]^

When local tissue injury is detected, inflammatory cells that are already present in the kidney and those that enter it cause programmed cell death (PCD) of TECs by generating and releasing multiple inflammatory cytokines and activating different pathway proteins. During the initial phases of AKI, M1 macrophages release several proinflammatory cytokines, which include interleukins and TNF‐α. These cytokines interact to specific receptors in tubular epithelial cells TECs, promoting PCD. This causes TECs separation, tubule obstruction, and function deterioration.^[^ ^32^ ^]^ In clinical practice, an elevated production of IFN‐α is often noted in kidney biopsies from patients experiencing AKI after kidney transplantation.^[^ ^29^ ^]^ Plasmacytoid dendritic cells (PDCs) are a unique subset of dendritic cells specialized in the production of interferon (IFN). PDCs can directly trigger apoptosis of TECs in vitro through IFN‐α production, indicating that PDCs contribute to AKI by promoting detrimental effects through IFN‐α.^[^ ^29^ ^]^ Furthermore, IL‐17A induces TEC apoptosis in AKI associated with sepsis.^[^ ^33^ ^]^

In summary, in AKI, damage to renal intrinsic cells causes circulating immune cells to infiltrate the body and macrophages to polarize, thereby enhancing renal inflammatory reactions. Furthermore, DCs and macrophages aid in the process of the programmed demise of TECs, contributing to tubular deactivation and ultimately resulting in a complex and dynamic microenvironmental state. Therefore, inhibiting the infiltration of immune cells or limiting the amplification of the inflammatory response could be an effective treatment strategy of AKI.

#### 2.1.2 ROS Damage

Elevated concentrations of ROS in the kidneys also aggravate the pathogenesis of AKI.^[^ ^34^ ^]^ Maintaining the redox equilibrium is essential for the restoration of kidney function, and ROS are key regulatory factors in AKI. Low levels of ROS are essential for normal renal oxidative metabolism, aiding in cell viability and growth, with no noticeable harmful impacts. Nevertheless, when the equilibrium of ROS between production and clearance is disturbed, excessive ROS from renal infiltration and/or intrinsic cells can lead to cellular apoptosis and/or necrosis through mitochondrial enlargement and disorder and deplete ATP production, thus damaging the kidney.^[^ ^35^, ^36^ ^]^ Excessive accumulation of ROS in damaged mitochondria attacks or modifies biomolecules (e.g., DNA, proteins, lipids), causing DNA damage, reducing organelle membrane stability and function, and interfering with signal transduction, ultimately leading to cell necrosis, apoptosis, and tissue inflammation.^[^ ^37^ ^]^ Subsequently, necrotic cells release proinflammatory factors such as TNFα, CCL2, IL‐1β, IL‐6, IL‐8, and transforming growth factor‐beta (TGF‐β). Inflammatory and chemotactic factors are involved in the chemotaxis and activation of leukocytes, leading to intensified cellular inflammation. Inflammatory damage also leads to structural injury of the tubulointerstitial space and microvascular endothelial dysfunction, resulting in increased permeability.^[^ ^38^ ^]^ Moreover, damaged microvascular endothelial cells interact with activated leukocytes and adhesion molecules, initiating an immune‐inflammatory cascade.^[^ ^39^ ^]^ Crucially, the continued stimulation of inflammatory effector cells and the subsequent discharge of multiple inflammatory mediators intensify the inflammatory response, causing additional harm to TECs and forming a detrimental cycle that eventually results in renal dysfunction.^[^ ^40^ ^]^ Therefore, efforts to clear excessive ROS in damaged tissues may aid in the recovery from AKI.

#### 2.1.3 Cell Death

Currently, cell death in AKI is generally categorized into apoptosis, pyroptosis, necrosis, and ferroptosis.^[^ ^35^, ^41^, ^42^, ^43^, ^44^ ^]^ The cell category, microenvironment, and injury phase in the kidney dictate the process of cell death. Multiple pathways of cell death are all throughout to be involved in the onset of AKI.

Recent evidence indicates that ROS production, mitochondrial dysfunction, and the stimulation of TNF‐α signaling participate in the initiation of cell apoptosis. Lipopolysaccharide (LPS) stimulation leads to elevated expression of TNF‐α and Fas ligand, initiating cell apoptosis through the activation of TNFR and following caspase impulses.^[^ ^45^ ^]^ In addition to TNF signaling, excessive ROS can trigger cell apoptosis, including by stimulating p53. Furthermore, the activation of B‐cell lymphoma‐2‐associated X (Bax) by ROS and/or p53 moves to the exterior membrane of the mitochondria, causing the release of cytochrome c, a potent inducer of cell apoptosis.^[^ ^46^ ^]^ Infiltration of immune cells, consisting of monocytes/macrophages, neutrophils, and T cells, characterizes endotoxin‐induced renal damage. During the progression of sepsis, macrophage depletion hinders the clearance of microbes, and excessive macrophage apoptosis may pose a risk of immunosuppression, leading to secondary infections or even mortality.^[^ ^47^ ^]^ Therefore, the apoptosis signaling pathway is intimately associated with AKI, suggesting that inhibiting apoptosis could be a viable approach in preventing and treating AKI.

Pyroptosis, which is induced by PAMPs, DAMPs, and inflammatory factors, is a crucial type of necrosis with programming related to the progression of septic shock and tissue injury.^[^ ^48^ ^]^ Pyroptosis results in the subsequent release of numerous DAMPs and pro‐inflammatory substances, which also results in the stimulation of inflammatory cells, thus intensifying the responses to inflammation and damaging different tissues.^[^ ^49^, ^50^ ^]^ M1 macrophages polarize under the stimulus of proinflammatory factors, including infection, endotoxins, and hypoxia. Upon activation with LPS, the TNF‐α/HMGB1 pathway is triggered in M1 macrophages, resulting in increased pyroptosis, whereas suppressing TNF‐α signaling can effectively inhibit macrophage pyroptosis.^[^ ^51^ ^]^ Both M1 and M2 macrophages contribute to the pathogenesis of AKI by modulating the production of inflammatory mediators and promoting cell death, including pyroptosis.

Programmed cell necrosis, also known as necroptosis, is noted in cisplatin, ischemia‐reperfusion, and nephrotoxicity caused by contrast agents. The initiation of Receptor‐Interacting Protein Kinase 1 (RIPK1), primarily by its binding with TNFR, is broadly considered to be a key mediator of necroptosis.^[^ ^52^ ^]^ RIPK1 further activates RIPK3 through the receptor homotypic interaction motif (RHIM), which then activates mixed lineage kinase domain‐like (MLKL).^[^ ^53^, ^54^, ^55^ ^]^ After activation, phosphorylated MLKL protein molecules aggregate into oligomers and move to the cell membrane, which results in cell swelling, bursting, and release of DAMPs.^[^ ^56^, ^57^ ^]^ Therefore, cell death is intimately associated with the prognosis of AKI, suggesting that inhibiting cell death could be a viable approach for its prevention and treatment.

#### 2.1.4 Renal Fibrosis

Mammalian renal tubular epithelia have a strong capacity for repair and regeneration to restore normal epithelial integrity after AKI.^[^ ^58^, ^59^ ^]^ Damaged epithelial and immune cells release several proinflammatory and profibrotic factors, aiding in the activation of resident fibroblasts and myofibroblasts, gradual accumulation of interstitial matrix proteins, irreversible scar formation, and gradual loss of functional renal units.^[^ ^60^, ^61^, ^62^ ^]^ The cell cycle encompasses the cell division stages G0, G1, S, G2, and M.^[^ ^63^ ^]^ During AKI, TECs cannot differentiate due to cell cycle arrest during G2/M, leading to the production and secretion of various profibrotic factors such as TGF‐β1 and connective tissue growth factor. Acting through paracrine mechanisms on peripheral cells and fibroblasts, these factors result in inadequate renal repair and fibrosis.^[^ ^62^, ^64^, ^65^ ^]^

Effective renal regeneration appears to require the resolution of macrophage‐mediated inflammation.^[^ ^66^ ^]^ Concerning the function of immune cells and the cell cycle, studies have indicated that M2 macrophages circumvent cell cycle arrest by generating the Wnt ligand Wnt7b, which targets damaged TECs, enhancing cell proliferation, repairing the kidney's tubular basement membrane, and preventing renal fibrosis.^[^ ^67^ ^]^ Macrophage migration inhibitory factor (MIF), a multipurpose cytokine in damaged tissue, is generated and secreted via macrophages in AKI and is considered an inherent protective element in the kidney.^[^ ^68^ ^]^ Following MIF knockout in sepsis and unilateral ureteric obstruction (UUO) mouse models, TECs experience G2/M cell cycle arrest, resulting in an amplified production of pro‐inflammatory and fibrotic mediators, exacerbating kidney fibrosis.^[^ ^69^ ^]^ Sirtuin 6 (SIRT6), a NAD‐dependent deacetylase, exhibits significant instability within macrophages and subjects to rapid proteasomal degradation.^[^ ^70^ ^]^ Research has shown that, in various AKI models, SIRT6 expression is reduced, and it mitigates kidney injury and exerts antioxidative effects by managing dynamics of mitochondria and restraining cell cycle arrest at the G2/M phase in TECs.^[^ ^71^, ^72^ ^]^ In addition, circulating bone marrow‐derived stromal cells can promote the repair process through paracrine effects mediated by microvesicles transferring proteins, receptors, mRNA, microRNAs, and organelles.^[^ ^73^ ^]^

### 2.2 Characteristics of the AKI Immune Microenvironment

**Figure 1:**
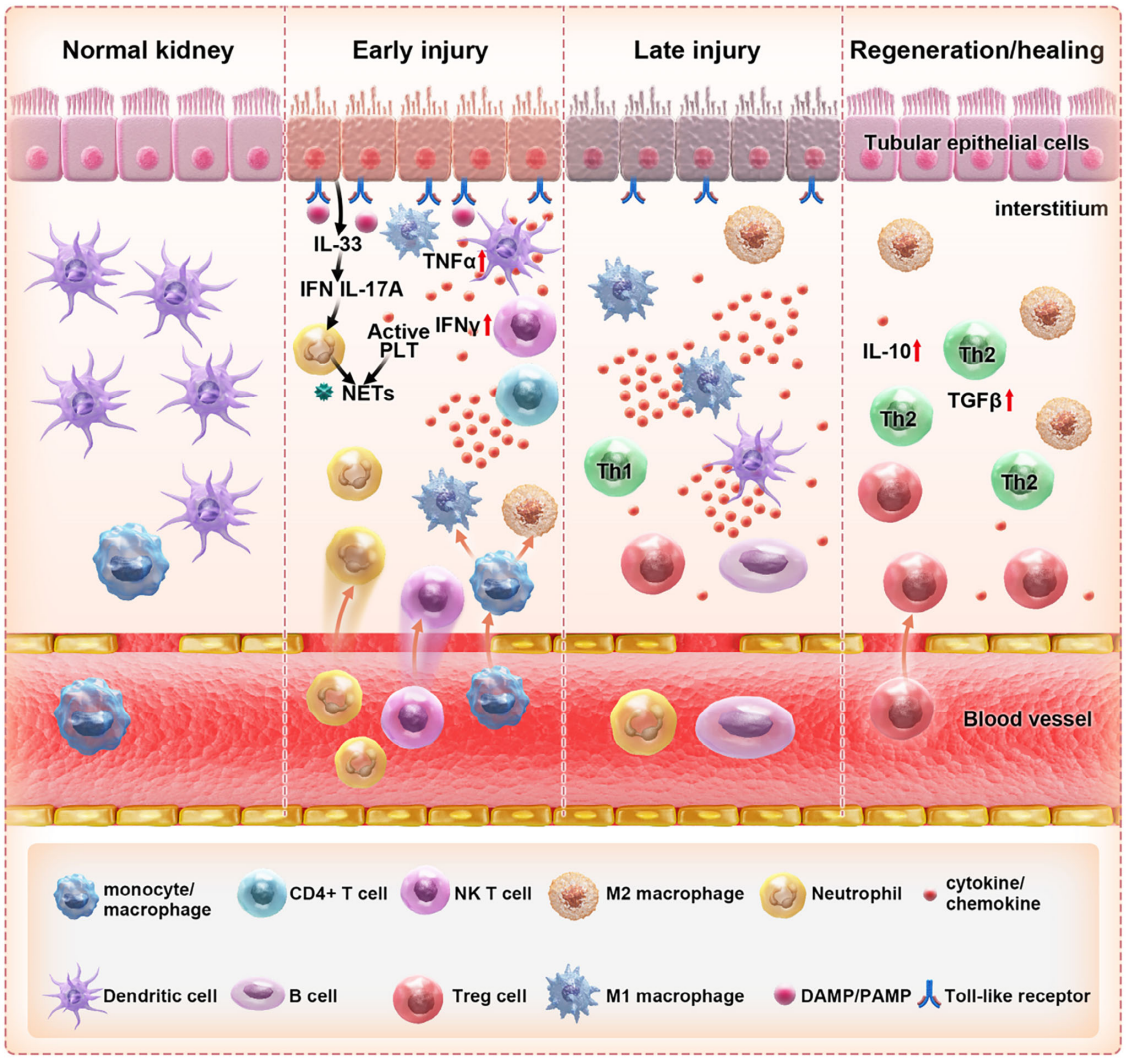
When the kidney experiences a sudden injury that is either aseptic or septic, certain molecules called danger‐associated molecular patterns (DAMPs) or pathogen‐associated molecular patterns (PAMPs) activate a response in the inflammasome, which leads to the recruitment of specific types of immune cells, mainly neutrophils, monocytes/macrophages, and NKT cells. Resident dendritic cells stimulate NKT cells to generate IFN‐γ and are the main source of TNF‐α. Dying tubular epithelial and endothelial cells produce DAMPs, stimulating neutrophil migration to the kidneys. Activated platelets collaborate with infiltrating neutrophils to create neutrophil extracellular traps (NETs), which exacerbate tissue damage. The cells of the innate immune system engage in interactions with the effector cells of the adaptive immune system. Collectively, they are accountable for the subsequent infliction of injury (plasma B cells and Th2 cells) and the restoration of tissue (M2 macrophages, Th1, and Treg cells).

The occurrence and development of AKI represents a complicated process that involves numerous immune cells and molecular elements (**Figure** **1**).^[^ ^1^, ^74^, ^75^ ^]^ Intrinsic immune and kidney cells, impaired by insufficient blood supply, oxygen, medications, and harmful substances, recruit additional immune cells through the secretion of chemokines. Intrinsic cells facilitate macrophage polarization, while immune cells contribute to various forms of programmed cell death, changes in intrinsic cell phenotypes, and disruption of the cell cycle, resulting in kidney damage.^[^ ^5^ ^]^ As AKI progresses, malfunctioning immune cells, such as lymphocytes and macrophages, result in a sustained inflammatory milieu. Consequently, a localized immune environment is formed, affecting renal tissue regeneration and leading to CKD.^[^ ^11^, ^76^ ^]^ The primary causes of poor AKI regeneration are heightened inflammatory responses from the ongoing infiltration of macrophages, T cells, and other immune cells, along with an increase in the aggregation and activation of adaptive immune cells. Immune cells exhibit high plasticity and diversity, participating in almost all events from kidney injury to repair and subsequent fibrosis. For example, macrophage phenotypes play different roles at various stages of injury, including pro‐inflammatory M1 cells during the injury phase and anti‐inflammatory M2 cells during the recovery phase.^[^ ^77^, ^78^ ^]^ Similarly, lymphocytes have multiple subtypes, ensuring precise and comprehensive regulation of the immune response in damaged kidneys.^[^ ^28^ ^]^ Upon activation by external (e.g., microbial antigens) or internal events, these cells generate inflammatory mediators that may cause kidney diseases, trigger regulatory responses intended to suppress inflammation, repair tissue damage, and regain internal balance. As an example, microbial antigens may attach to innate immune receptors on dendritic cell surfaces, causing these cells to release certain chemokines and cytokines.^[^ ^79^, ^80^ ^]^ and migrate to lymph nodes, where they enhance the immunological response by stimulating T lymphocytes (Th1 CD4+, and cytotoxic cells). Therapeutically, reshaping the immune microenvironment of AKI by suppressing inflammatory reactions and decreasing cytokine production is crucial. A harmonized immune microenvironment, with innate and adaptive immune regulation might achieve more advantageous clinical treatment results. Thoroughly grasping the potential cellular‐molecular mechanisms offers invaluable insights for devising focused clinical interventions.

#### 2.2.1 Macrophages

Macrophages are crucial in mediating kidney damage, promoting the healing process, and scarring in the kidneys after an injury. In normal kidneys, macrophages are infrequent, but their quantity significantly rises within 24 h following damage.^[^ ^81^ ^]^ Upon being mobilized to a damaged kidney, monocytes undergo a transformation into macrophages, which exhibit functional diversity and are capable of being activated through either the M1 or M2 pathways. Macrophages, as part of the innate immune system, have an essential role in the initial reaction to infections. As previously stated, infection leads to the migration and infiltration of monocytes into organs, where they transform into pathogenic or tissue‐repair phenotypes through the M1 or M2 pathway. The functional inclination of the monocyte/macrophage group is contingent on the inflammatory tissue microenvironment.

M1 macrophages are triggered through their incorporation of DAMPs and PAMPs to PRRs and the interferon‐γ secreted by T‐helper cells and NKT cells. M1 macrophages generate chemokines and inducible nitric oxide synthase (iNOS). The latter combines with ROS, resulting in the production of cytotoxic peroxynitrite.^[^ ^82^ ^]^ Activated M1 macrophages additionally generate many proinflammatory cytokines, thereby fueling the inflammatory stage of kidney injury.

M2 macrophages are macrophages that are involved in wound healing and immune regulation. The reasons for macrophages transitioning from a pro‐inflammatory state to a reparative state are unclear, but may be associated with the anti‐inflammatory functions of macrophages and alterations within the microenvironment at the injury location causing transcriptional shifts in resident macrophages. Wound‐healing macrophages generate extracellular matrix (ECM), but when this process becomes dysregulated, it can lead to tissue fibrosis.^[^ ^83^ ^]^ Immune‐regulating macrophages are tasked with producing IL‐10 and TGF‐β, serving anti‐inflammatory roles and mitigating the immune reaction to tissue injury.^[^ ^78^ ^]^ However, during severe or repeated kidney injury, damaged renal tubular cells release chemokines, promoting M2 macrophages to enter the kidney via selectins, integrins, and transendothelial migration. These M2 macrophages continually produce growth factors/cytokines, such as TGF‐β1 and epidermal growth factor (EGF), which induce activation of interstitial fibroblasts, leading to excessive ECM deposition and the progression of kidney fibrosis.^[^ ^84^ ^]^ Therefore, regulation of the M1/M2 phenotype balance represents a critical mechanism in the treatment of AKI.

#### 2.2.2 T Cells

CD4+ and CD8+ T cells are the two main types of T lymphocytes involved in cellular immunity. Upon activation, initial CD4+ T cells diversify into helper T cells (Th) 1, 2, 17, or regulatory T cells. CD8+ T cells can proliferate and transform into cytotoxic effector cells, producing IFN‐γ and TNF‐α, responding to pathogens, and migrating to eliminate infections. During infection, CD8+ T cells are sensitized by antigen‐presenting cells in the lymph nodes and spleen.^[^ ^85^, ^86^ ^]^

Th1 cells generate IFN‐γ and TNFα, which are two important proinflammatory cytokines, whereas Th2 cells secrete anti‐inflammatory cytokines, such as IL‐13, IL‐10, IL‐4, and IL‐5.^[^ ^86^ ^]^ Studies indicate that the Th1 inflammatory cell pattern is pathogenic, leading to aggravated kidney injury, whereas the Th2 cell pattern is protective in renal ischemia‐reperfusion injury (IRI) models.^[^ ^85^ ^]^ Tregs are a subset of CD4+ cells defined by the presence of the CD25 receptor. Research indicates that Tregs mitigate AKI.^[^ ^87^ ^]^ After kidney injury, Tregs suppress adaptive immune responses in several ways, including by secreting inhibitory cytokines that bind to Foxp3^−^ T cells and inhibit their function, leading to cell cycle arrest or apoptosis; attaching to DCs and inhibiting their maturation; and attaching to innate immune system effectors to reduce their capacity for co‐stimulation or antigen presentation.^[^ ^88^ ^]^ Depleting Tregs using anti‐CD25 antibodies (PC61) in ischemic AKI animal models has been shown to lead to worsening of AKI by increasing T cell proliferation and cytokine levels, aggravating renal tubular injury, and reducing the tubular recovery rate. This is thought to be a result of IL‐10‐mediated inhibition of the innate immune system.^[^ ^88^ ^]^ Moreover, Jaworska and colleagues observed improvement in IRI following Treg transfer, an effect dependent on the expression of programmed death ligands 1 and 2 (PD‐L1/2) by Tregs.^[^ ^89^ ^]^ Therefore, increasing the number of renal Tregs and enhancing Treg activity represent novel targets for AKI immunotherapy.

#### 2.2.3 NK T Cells, DCs, and Other Cells

NK T cells play a crucial role in the immune system, possessing diverse capabilities such as combating tumors, viruses, bacteria, infections, and regulating immune responses.^[^ ^90^, ^91^ ^]^ NK T cells can directly identify antigens on CD1d molecules from antigen‐presenting cells and are thus classified as CD1d‐restricted T cells. It has been reported that NK T cells infiltrate the kidney at a very early stage after ischemia.^[^ ^92^ ^]^ Given that NK T cells can be rapidly activated, leading to the production of cytokines (including IFN‐γ and IL‐17) and recruitment of innate immune cells,^[^ ^93^ ^]^ they are considered to be the main effector and regulatory cell type in the very early stages of IRI. NK T cells are thought to primarily mediate detrimental effects on ischemic AKI.^[^ ^93^ ^]^ However, the protective role of NK T cells in renal IRI has also been reported, and it is believed that the extent of renal injury may affect the function of NK T cells.

DCs are a type of mononuclear phagocyte, which, as resident cells in multiple organs, play various roles and act as connectors between innate and adaptive immunity. Within the kidneys, dendritic cells constitute a diverse group, executing various functions including antigen presentation, T cell stimulation, and IL‐10 secretion.^[^ ^94^ ^]^ Kidney dendritic cells are situated between renal tubules and peritubular capillaries, allowing interactions with effector cells, endothelial cells, and epithelial cells. In AKI, renal DCs are activated by binding to cell debris and other mediators through TLRs and NLRs, leading to increased expression of co‐stimulatory molecules and pro‐inflammatory cytokines (e.g., TNFα) and presenting antigens to NK T cells in draining lymph nodes, thus promoting NK T cell‐mediated inflammation.^[^ ^79^, ^80^ ^]^ Blocking the interaction between renal DCs and NK T cells and inhibiting IRI‐induced renal inflammation and NK T cell activation can protect the kidneys from IRI.

In summary, the occurrence, development, and repair of AKI are complex processes involving a variety of immune cells and molecules, each playing different roles at various stages of AKI. The influence of these cells and molecules on AKI depends on their subtypes, and is additionally regulated by localized signals present in the microenvironment. Acquiring a comprehensive understanding of the functions of these immune cells in various types of AKI injuries is essential for pinpointing potential treatment targets. As a result, strategies focused on modulating kidney immune responses and reforming the local inflammatory microenvironment represent promising methods for facilitating AKI recovery. These strategies include modulating macrophage polarization, promoting the differentiation of T cells into regulatory T cells, and enhancing DC tolerance to augment, regulate, or suppress relevant immune responses. Future research efforts should integrate insights from various injury models and potential clinical conditions to fully explore the potential of immune cells and the immune microenvironment in treating AKI.

### 2.3 Types of IMNs in AKI Treatments

Currently, several strategies have been developed to specifically target the harmful immune environment that causes AKI, with the aim of reducing its severity. However, these strategies are not ideal, primarily because of the complex immune microenvironment of the kidneys and the lack of renal targeting. Immunomodulatory nanosystems, with their superior physicochemical properties, effective drug‐carrying capabilities, and good biocompatibility, have become a promising approach for treating AKI. In the past 20 years, a significant number of polymer, lipid, metal, and hybrid nanoparticles have been developed as efficient drug delivery tools for modulating the AKI immune microenvironment.

In the development of IMNs for treating AKI, researchers are actively studying their renal targeting ability to reduce the side effects of systemic administration. However, targeted nanoparticles have achieved limited success in clinical medicine because of to a few obstacles faced by systemic administration, which may rely on the surface properties of the nanoparticles. First, nanoparticles usually cannot efficiently escape the vascular system to access extravascular targets,^[^ ^95^ ^]^ resulting in significant retention in the liver and spleen, where they are gradually engulfed over time, thereby preventing their arrival at target cells.^[^ ^96^ ^]^ Researchers have integrated particular targeting ligands with nanomaterials to improve renal location by functioning as intelligent devices that are responsive to the surrounding conditions.^[^ ^97^ ^]^ Second, upon interaction with serum proteins, nanoparticles promptly develop a protein corona,^[^ ^98^ ^]^ that can effectively conceal the targeting ligands and diminish their ability to target specific sites within the body.^[^ ^95^ ^]^ Nanomaterials can be surface‐modified or coated to reduce the formation of the protein corona, thereby enhancing their biocompatibility and facilitating renal clearance. For example, biocompatible hydrophilic polymers are usually used to encapsulate nanoparticles to reduce endocytosis, prevent plasma protein adsorption, and thus prolong the circulation half‐life. Zwitterionic ligands and coating with polyethylene glycol are two widely used surface modification methods to produce nanoparticles with neutral surface charges, allowing for renal clearance.^[^ ^95^ ^]^ Finally, for some organs, such as kidneys or brain, there are additional challenges to drug delivery carriers beyond optimizing blood circulation. In the kidneys, the glomerular filtration barrier presents a major challenge that drug delivery systems need to overcome. Scientists have thoroughly investigated the ways in which nanosystems are removed from the body using laboratory experiments and studies conducted on living organisms.^[^ ^99^ ^]^ Advanced diagnostic methods such as imaging, biodistribution analysis, and histological examination aid investigators in observing the function of nanosystems across diverse tissue and organ systems.^[^ ^99^ ^]^ Hence, taking these factors into account and performing thorough preclinical and clinical assessments to ascertain the security and biocompatibility of immunonanosystems for AKI treatment is essential.

**Figure 2:**
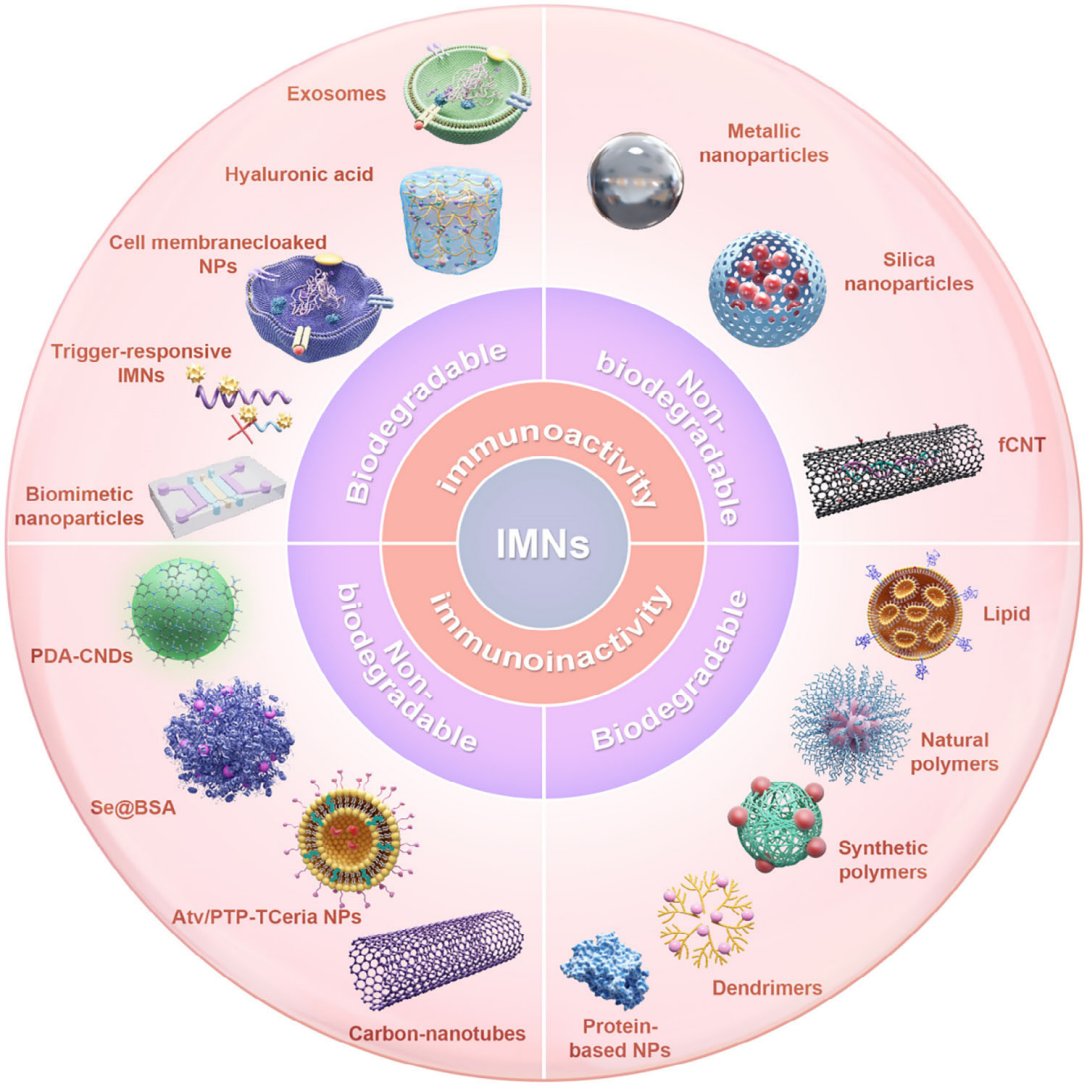
IMNs are classified by biodegradability and immunological reactiveness into four categories. The first category comprises biologically‐derived materials that exhibit immunomodulatory action and biodegradability, including cell membrane‐derived nanoparticles, extracellular vesicles, and exosomes. The second category includes IMNs made from non‐biodegradable nanomaterials with immunoactivity, such as metal nanoparticles (Au, Ag, Cu, FeO_2_) and acid‐treated and carboxyl functionalized carbon nanotubes. The third category of IMNs includes biodegradable, immunologically inert nanoparticles of lipids, polymers, and dendrimers. The fourth category of IMNs includes non‐functionalized CNTs, triphenylphosphine (TPP)‐modified Ceria NPs coated with ROS‐responsive organic polymer (mPEG‐TK‐PLGA) and encapsulated with atorvastatin (Atv/PTP‐TCeria NPs), and selenate, ascorbic acid, and bovine serum albumin (BSA)‐prepared Se@BSA NPs.

IMNs can be generally classed into four categories according to the biodegradability and the immunological reactiveness (**Figure** **2**). The first category comprises biologically‐derived materials that exhibit immunomodulatory action and biodegradability. These materials include nanoparticles derived from cell membranes,^[^ ^100^ ^]^ extracellular vesicles,^[^ ^101^ ^]^ and exosomes.^[^ ^102^ ^]^ The second category comprises IMNs that are non‐biodegradable and possess established immunoactivity. This category includes metal nanoparticles (silver [Ag], gold [Au], copper [Cu], iron oxide [FeO_2_]) and acid‐treated and carboxyl functionalized‐carbon nanotubes (f‐CNTs). The third classification of IMNs comprises biodegradable and immunologically inert materials, specifically nanoparticles made of lipids, polymers, and dendrimers. The nanoparticles function as carriers of immunomodulatory molecules, facilitating their controlled delivery to the kidneys for the treatment of AKI. These particular immunomodulating nanosystems help transport immunomodulators to the injured kidney. However, these nanocarriers may cause unintended immune responses because the drug is released uncontrollably at sites that are not the intended target. Lastly, the fourth category of IMNs consists of non‐biodegradable and immunologically inert materials. This category includes non‐functionalized carbon nanotubes (CNTs), triphenylphosphine (TPP)‐modified Ceria nanoparticles (NPs) coated with ROS‐responsive organic polymer (mPEG‐TK‐PLGA) and simultaneously encapsulated with atorvastatin (Atv/PTP‐TCeria NPs)^[^ ^103^ ^]^ and Se@BSA NPs,^[^ ^104^ ^]^ which are prepared with selenate, ascorbic acid, and bovine serum albumin (BSA).

### 2.4 Mechanisms of IMNs in AKI Treatment

Functionally, the IMNs used for AKI treatment utilize various immunomodulatory mechanisms, which can generally be categorized into seven types (**Table** **1**). In this part, we will review the recent progress in AKI treatment strategies involving nanosystems for immune microenvironment modulation and talk about the limitations of these functional nanosystems.

**Table 1:** Nanosystem‐based strategies for immunomodulation in AKI.

Mechanisms	Strategies	Example	Details
**Reduction of pro‐inflammatory cytokine production**	Delivery of anti‐inflammatory drugs	PEGylated liposomal prednisolone^[^ ^112^ ^]^	Interaction with macrophages reduces IL‐6 production and pro motes glucocorticoid receptor translocation to downregulate CCL2.
		Dsp‐loaded SA‐NPs^[^ ^109^ ^]^	Compared with free Dsp solution, Dsp‐loaded SA‐NPs significantly improve kidney function, reduce production of pro‐inflammatory factors, and regulate oxidative stress factors and apoptotic proteins.
		Kim‐1 antibody‐conjugated PLGA NPs^[^ ^113^ ^]^	Reduced IL‐1β production by targeting injured kidney cells with an antibody that recognises Kim‐1 expression in proximal tubular cells.
	Gene therapy	chitosan/COX‐2 siRNA NPs^[^ ^117^ ^]^	Decreased mRNA production of proinflammatory factor. (e.g., TNF‐α and IL‐6)
	Delivery of EVs	Exosome‐based delivery of super‐repressor IκBα^[^ ^174^ ^]^	SRIκB is efficiently encapsulated and transported to neutrophils and macrophages via EVS, reducing inflammation by suppressing NF‐κB signaling in septic and ischemic kidney injuries respectively.
	Delivery of thrombin inhibitors	perfluorocarbon nanoparticles (PFC NP)^[^ ^175^ ^]^	PFC nanosystems can block thrombin signaling and microvascular thrombosis formation, limit the amplification of inflammatory signals, protect renal tubular microstructures, and mitigate damage in acute kidney injury models.
**Neutralizing Overproduced Cytokines**	Delivery of cytokine neutralizing antibody	SAP/Hep loaded anti‐TNF‐α and HGF^[^ ^118^ ^]^	Lower renal tissue pro‐inflammatory cytokines, macrophage infiltration, and serum biomarkers.
	Delivery of DNA nanodevice	ac5a‐rDNAs^[^ ^119^ ^]^	aC5a‐rDONS demonstrated the ability to clear ROS in the early stages of I/R and inhibit the inflammatory response in the later stages of I/R.
**Regulating immune cell infiltration**	Reprogram macrophage phenotype	nHA/PLBR^[^ ^124^ ^]^	The proportion of CD68+ macrophages in the kidneys of AKI‐damaged rats treated with nHA/PLBR significantly decreased.
		SAP‐MT^[^ ^127^ ^]^	Reduced macrophage infiltration and proinflammatory marker expression compared to free drug.
	Deliveryof chemokine receptor antagonists	linear chitosan chemokine receptor CXCR4 antagonist (PCX)^[^ ^6^ ^]^	In AKI models with cisplatin and bilateral ischaemia reperfusion injury, this chitosan can transfer siP53 to the kidneys and inhibit CXCR4 and P53 gene silencing.
	Gene therapy	mir‐I‐LV^[^ ^111^ ^]^	Compared to lipopolysaccharide, mir‐I‐LV significantly reduced the expression of renal mir155, leading to a marked reduction in inflammatory cell infiltration.
**Modulating macrophage polarization**	Delivery of antioxidative drugs	Curcumin‐carrying nanoparticles(Cur‐NPs)^[^ ^130^ ^]^	Curcumin relieved cisplatin‐induced kidney inflammation through inhibiting Mincle‐maintained M1 macrophage phenotype.
	Delivery of stem cells	Exosomes derived from mesenchymal stem cells^[^ ^126^ ^]^	MSC‐exo with high CCR2 expression could reduce the concentration of free CCL2, inhibiting its function to recruit or activate macrophages.
	Delivery of anti‐inflammatory drugs	PDA@MF NPs^[^ ^133^ ^]^	PDA@MF NPs successfully reprogrammed M1 macrophages into M2 macrophages, synergistically scavenging ROS and producing O_2_.
	Promoting the expression of IL‐33	CSPB NPs^[^ ^134^ ^]^	CSPB NPs increase IL‐33 expression, which polarises M1‐type macrophages to anti‐inflammatory M2‐types that decrease pro‐inflammatory factors.
**Supplementation with cytokines**	Delivery of Evs	IL‐10+EV_S_^[^ ^101^ ^]^	Because Evs' integrins and adhesion components target renal tubulointerstitial macrophages and renal tubular epithelial cells, IL‐10+EV_S_ can reduce renal ischemia/reperfusion (I/R) injury.
			
	Delivery of DNA Nanoraft	DON+IL‐33^[^ ^141^ ^]^	IL‐33 from Nanoraft's prolonged retention and release in the kidneys swiftly expands ILC2s and Tregs, treating ischaemic AKI.
**Regulating oxidative stress**	Delivery of RONS scavenge	TFNAs^[^ ^143^ ^]^	TFNAs serve as a strong scavenger of ROS, are preferentially absorbed by the kidneys, restoring renal function in models of rhabdomyolysis‐induced AKI.
			
	Delivery of antioxidant nanoenzymes	Ti_3_C_2_ MXene nanosheets (TPN)^[^ ^145^ ^]^	TPN clear ROS by accepting electrons from ROS.
		Platinum NPs^[^ ^149^ ^]^	Platinum nanoparticles mimic multiple enzymes and scavenge broad‐spectrum ROS.
	Delivery of ROS‐responsive drug	SC‐TK‐SS31^[^ ^151^ ^]^	SC‐TK‐SS31 achieves precise targeting of ROS.
	Delivery of Nrf2 activator	PLGA‐OltiPraz NPs^[^ ^152^ ^]^	PLGA scavenges ROS via Nrf2 signalling.
**Modulating tolerogenic immune response**	Delivery of anti‐inflammatory drugs	SNP@TEC^[^ ^100^ ^]^	SNP@TEC not only directs macrophages toward an anti‐inflammatory M2 phenotype, but also inhibits T cell activation and induces T cells to differentiate into regulatory T cells by blocking CD80 on dendritic cells.
			
	Modulating the expression of cytokines	MeNP4^TP[^ ^156^ ^]^	MeNP4^TP^ releases TP to inhibit macrophages and DCs and generate regulatory T cells.

#### 2.4.1 Reduction of Pro‐Inflammatory Cytokine Production

During AKI, renal immune and intrinsic cells produce numerous pro‐inflammatory cytokines, namely IL‐6, IL‐8, IL‐1β, TNFα, and TNF‐γ, as well as anti‐inflammatory cytokines at the site of inflammation, such as IL‐4, IL‐10, IL‐13, and TGF‐β. The overproduction of pro‐inflammatory cytokines can prolong inflammation, worsening renal fibrosis. In comparison to other types of renal protective medications, the application of IMNs with anti‐inflammatory drugs in improving AKI treatment has been extensively explored, including steroids, innovative oligonucleotide‐based therapies, and organically generated compounds, all of which have been integrated into different structural categories of IMNs and assessed in numerous in vivo AKI models. Generally, the targeted delivery of anti‐inflammatory medications to the local tissues of the damaged kidney can diminish the generation of pro‐inflammatory cytokines^[^ ^105^ ^]^ and the expression of TLRs 1–4, 6, and 9, as well as complement receptors 3 and 5a.^[^ ^106^, ^107^, ^108^ ^]^ Because macrophages can actively uptake large particles, they serve as the main target for localized anti‐inflammatory treatments involving liposomes that contain glucocorticoids (GCs).^[^ ^109^ ^]^ The size of liposomes (100–500 nm) aids in their local uptake by inflamed tissues, as the liposomes use the enhanced endothelial permeability within inflamed tissues for extravasation, which does not occur through the normal endothelium in healthy tissues. Furthermore, PEGylation of liposomes prolongs their circulation time, allowing the effective payload to persist until extravasation, where interaction with macrophages reduces IL‐6 production and promotes glucocorticoid receptor translocation to downregulate CCL2, leading to a shift in the kidney toward a less pro‐inflammatory state. MicroRNAs (miRNAs) are endogenous non‐coding RNA molecules that target mRNA (gene regulation) and affect translation. Among them, mir‐155 actively participates in the initiation of inflammation within hepatocytes and significantly contributes to kidney damage through the regulation of immune responses. Mir‐155 is also involved in the effects on renal IRI through the regulation of FOXO3a.^[^ ^110^ ^]^ Chen et al.^[^ ^111^ ^]^ prepared mir‐155 inhibitor liposome microvesicles (mir‐I‐LV) for the efficient treatment of AKI. The results showed that compared to LPS, mir‐I‐LV significantly lowered the expression of renal mir155, leading to a marked reduction in inflammatory factor expression, inflammatory cell infiltration, and significant mitigation of renal pathological changes. Resveratrol is a natural polyphenolic compound with unique anti‐inflammatory effects, which can shift the inflammatory stage of AKI toward active repair and regeneration. For example, continuously administering resveratrol for 14 days before renal IRI can diminish the secretion of pro‐inflammatory cytokines in rats.^[^ ^112^ ^]^ However, its unfavorable pharmacokinetic characteristics limit its clinical application. Consequently, researchers have developed Kim‐1 antibody‐conjugated poly(lactic‐co‐glycolic acid)(PLGA)nanoparticles encapsulating resveratrol, utilizing antibodies that recognize Kim‐1 expressed on proximal renal tubular cells for specific targeting of damaged kidney cells to lessen the production of pro‐inflammatory cytokines.^[^ ^113^ ^]^

COX‐2 is a well‐known pro‐inflammatory enzyme that is induced by inflammatory stimuli.^[^ ^114^ ^]^ Specific COX‐2 inhibitors are believed to prevent renal injury and apoptosis in the UUO model.^[^ ^115^ ^]^ However, COX‐2 inhibitors can cause side effects such as impaired glomerular filtration rate, peripheral edema, and hypertension, which may increase the risk of cardiovascular complications. Based on these findings, therapeutic intervention in COX‐2 expression can be considered a new treatment method to prevent or reduce renal damage, apoptosis, oxidative stress, and inflammation in the UUO model. RNA interference, mediated by small interfering RNA (siRNA) that suppresses pro‐inflammatory cytokines at the mRNA level, represents an alternative anti‐inflammatory therapeutic strategy. Therapeutic approaches using siRNA have been developed for various clinical conditions, including AKI.^[^ ^116^ ^]^ Yang et al.^[^ ^117^ ^]^ developed chitosan/COX‐2 siRNA nanoparticles targeting cyclooxygenase‐2 to alleviate renal injury caused by unilateral ureteral obstruction in mice. Leveraging macrophages’ homing traits, COX‐2 siRNA can particularly accumulate in the injured kidney, diminishing the manufacturing of IL‐6 and TNF‐α mRNA, so only a small quantity of siRNA is needed to achieve therapeutic effects.

#### 2.4.2 Neutralizing Overproduced Pro‐Inflammatory Factors

**Figure 3:**
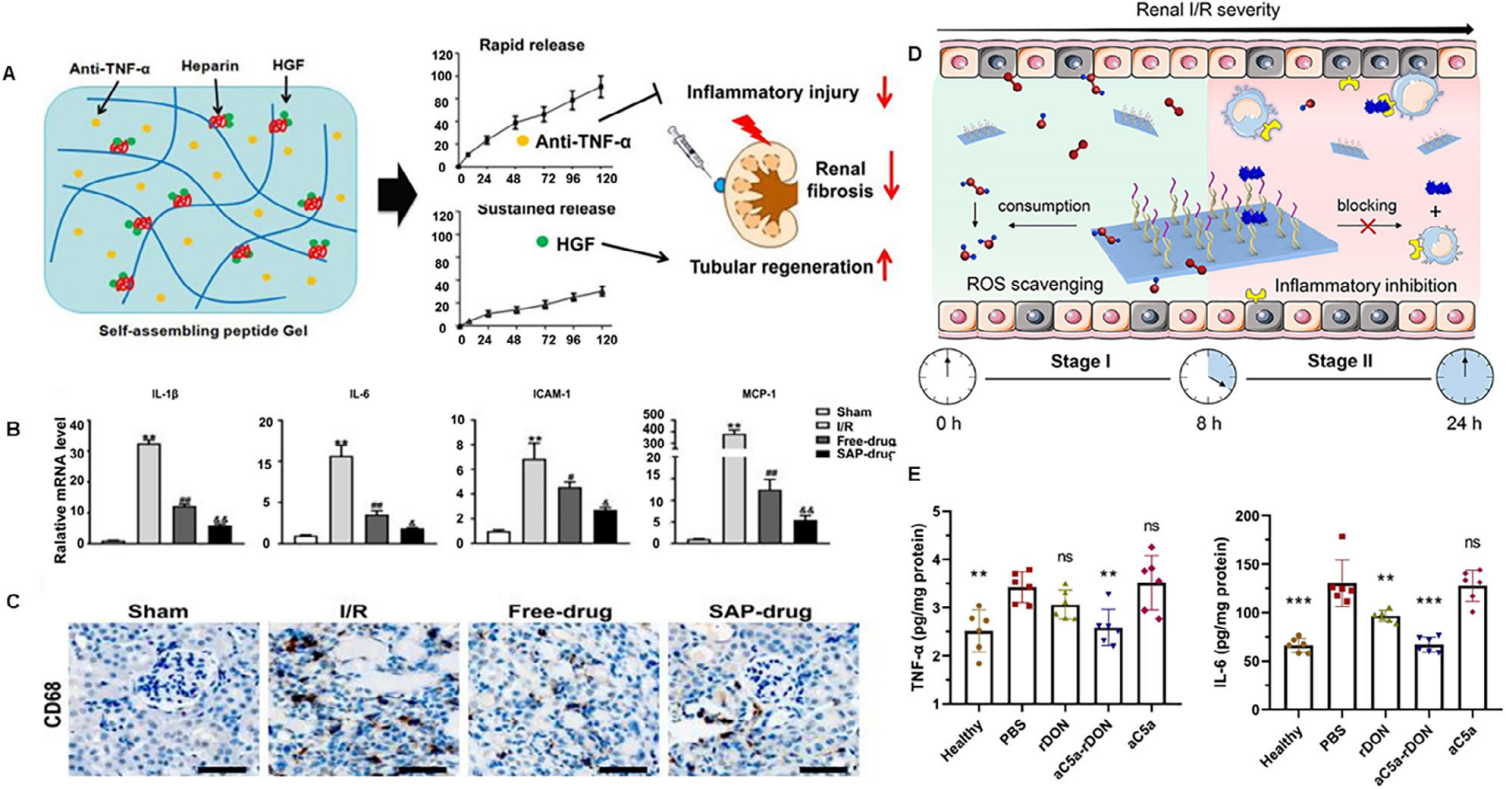
A) Dual‐drug delivery design (KLD2R/Hep hydrogel) for promoting kidney repair. B) Real‐time PCR analysis of IL‐1β, IL‐6, ICAM‐1, and MCP‐1 genes in the kidneys at 3 days after injury. C) Micrographs of CD68 IHC staining in the kidneys. Reproduced with permission.^[^ ^118^ ^]^ Copyright 2020, Elsevier. D) aC5a‐rDONs enabled dual‐drug combination therapy for sequential protection of the kidney in multiple stages of AKI. E) TNF‐α and IL‐6 levels in kidney homogenates of each group. Reproduced with permission.^[^ ^119^ ^]^ Copyright 2021, American Chemical Society.

The working principle of the second type of IMN is to isolate or neutralize (block) the excessively produced pro‐inflammatory cytokines in the inflammatory microenvironment. Reducing the level of proinflammatory cytokines decreases their binding to similar receptors on target cells, disrupting the cytokine cascade. IMNs primarily accomplish this by anti‐cytokine agents, such as monoclonal antibodies and anti‐complement component adaptors. Liu et al.^[^ ^118^ ^]^ co‐loaded TNFα neutralizing antibodies and hepatocyte growth factor into hydrogels to repair renal tissue in AKI. The hydrogel, composed of self‐assembling peptides and heparin (**Figure** **3**A), rapidly released TNFα neutralizing antibodies because of the strong attraction between hepatocyte growth factor and heparin, while continuously releasing hepatocyte growth factor. In an IR‐induced AKI mouse model, compared to free drugs and blank hydrogel, the co‐loaded hydrogel with local injection into the renal capsule offered better protection, increased serum biomarkers, improved renal tubular regeneration, and decreased infiltration of proinflammatory cytokines (Figure 3B) and macrophages (Figure 3C) in renal tissue. Chen et al.^[^ ^119^ ^]^ selected a DNA‐based novel nanodevice as a smart nanomedicine for new drug development based on the pathogenesis and staging of AKI (Figure 3D). Specifically, *N*‐Acetyl‐L‐cysteine (NAC), an antioxidant used clinically to clear intracellular ROS and prevent oxidative stress, and a C5a antagonist that can compete with C5a for high‐affinity binding to C5aR, were used to inhibit the C5a‐mediated inflammatory response. Combining the therapeutic effects of these two drugs, aC5a‐rDONS demonstrated the ability to clear ROS in the early stages of IRI and inhibit the C5a‐mediated inflammatory response (Figure 3E) in the later stages of IRI, achieving sequential treatment of AKI.

#### 2.4.3 Regulating Immune Cell Infiltration

**Figure 4:**
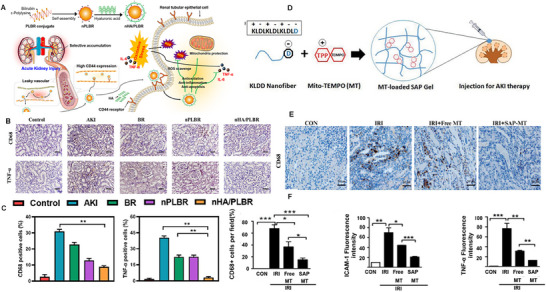
A) Chemical structure of nHA/PLBR and schematic illustration showing the relief of AKI by (nHA/PLBR). B,C) Quantitation of CD68+ and TNF‐α+ cells from the five groups. Reproduced with permission.^[^ ^124^ ^]^ Copyright 2021, Elsevier. D) Design and characterization of the cationic KLDD peptide. E) Micrographs of IHC staining for CD68 of the kidneys at day 5 after IRI. F) Quantification of CD68+ macrophages, ICAM‐1, and TNF‐α protein levels detected by IHC using Image ProPlus analysis. Reproduced with permission.^[^ ^127^ ^]^ Copyright 2018, Taylor & Francis.

Macrophages migrate to the damaged kidney during the progression of AKI, leading to renal structural damage and secretion of inflammatory factors, thus worsening inflammation and swiftly diminishing renal function. Because of the rarity of CD68+ cells in healthy kidneys, their presence within renal tissue likely results from monocytes migrating from the circulation post‐injury. In the renal tissue of rats with an AKI model, researchers noted an increased level of CD68+ macrophage infiltration.^[^ ^120^ ^]^ Stocker et al. initially verified the defensive impact of bilirubin against oxidative injury.^[^ ^121^ ^]^ Subsequently, its potent antioxidant and anti‐inflammatory properties were unveiled, garnering increased interest.^[^ ^122^ ^]^ However, bilirubin's insolubility in water at physiological pH hinders its potential for AKI treatment. Huang et al.^[^ ^123^ ^]^ synthesized a polylysine‐bilirubin conjugate (PLBR) for islet protection, demonstrating bilirubin's capacity to steer macrophages toward an M2 phenotype. Intriguingly, PLBRs can self‐assemble into nanoparticle form (NPLBR) and still possess strong antioxidant and anti‐inflammatory characteristics (**Figure** **4**A). Compared to the bilirubin or nPLBR group, the proportion of CD68+ macrophages and TNF‐α in the kidneys of AKI‐damaged rats treated with hyaluronic acid (HA) coated ε‐polylysine‐bilirubin conjugate (PLBR) nanoparticle (nHA/PLBR) was significantly decreased (Figure 4B,C).^[^ ^124^ ^]^ This indicates that bilirubin has a significant protective role in AKI by reducing monocyte infiltration. However, this study was limited to testing the rat model of ischemia‐reperfusion‐induced AKI. Its efficacy should be further explored in future studies using more advanced animal models or even in clinical patients. In addition to bilirubin, Mito‐Tempo (MT) is an ordinary mitochondrial‐targeted antioxidant consisting of 2,2,6,6‐tetramethylpiperidine‐N‐oxyl (Tempo) and triphenylphosphonium cation (TPP+).^[^ ^125^ ^]^ However, the clinic application of MT is significantly hindered by its brief duration of action and adverse effects within the living organism. It has been revealed that higher doses of MT do not have renal protective effects in AKI mice,^[^ ^126^ ^]^ as accumulated TPP+ can depolarize mitochondria.^[^ ^125^ ^]^ Therefore, the development of a slow‐release carrier for MT could reduce its side effects and improve its efficacy in treating AKI. Zhao et al.^[^ ^127^ ^]^ designed a lysine‐dendritic aspartic acid (KLDD), which could self‐assemble into crosslinked nanofiber hydrogels (SAP‐MT), with a release rate lower than that of free MT and KLD hydrogels (Figure 4D). SAP‐MT significantly reduces the level of CD68+ macrophage infiltration and TNF‐α in IRI mice (Figure 4E,F). In addition, chemokines secreted by neutrophils from reperfused blood lead to the recruitment of more inflammatory cells, thereby mediating kidney damage.^[^ ^23^ ^]^ The chemokine receptor, CXCR4, is involved in the migration of inflammation‐related cells associated with injury repair in AKI.^[^ ^23^ ^]^ Therefore, the application of chemokine receptor CXCR4 antagonists can reduce the infiltration of inflammatory cells.^[^ ^128^ ^]^ Researchers synthesized a linear chitosan chemokine receptor CXCR4 antagonist (PCX) through the Michael addition copolymerization of a benzene derivative with hexamethylene diacrylamide. This chitosan can also transfer siP53 directly to the kidneys of AKI mouse models that have been subjected to cisplatin and bilateral ischemia‐reperfusion injury, in which AKI is treated by P53 gene silencing and inhibiting CXCR4 to improve renal function and reduce renal damage.^[^ ^6^ ^]^

#### 2.4.4 Modulating Macrophage Polarization

**Figure 5:**
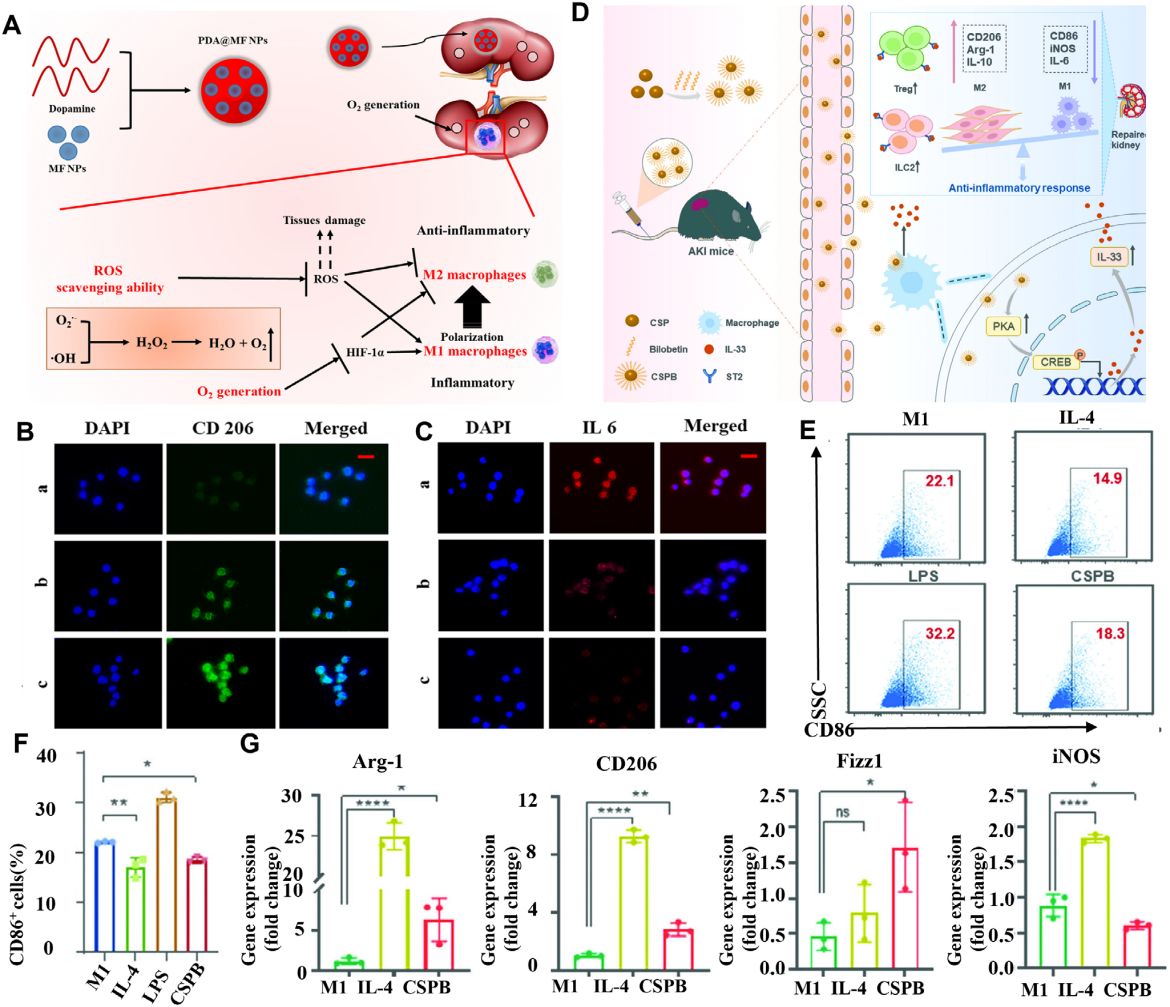
A) ROS‐related disease treatment schematic with PDA@MF NPs. B) PDA@MF NP therapy up‐regulated the level of CD206(M2 type marker). C) PDA@MF NP therapy down‐regulated the level of IL6 (pro‐inflammatory M1 type marker). Reproduced with permission.^[^ ^133^ ^]^ Copyright 2022, Elsevier. D) AKI mouse treatment with CSPB NPs activates IL‐33/ST2 and improves the immune microenvironment. E,F) The proportion of M1 macrophages (CD86 positive) cultured under different incubation conditions was analyzed by flow cytometry. G) qRT‐PCR analysis of Arg‐1, CD206, Fizz1, and iNOS in M1‐like macrophages treated with or without CSPB NPs for 24 h. Reproduced with permission.^[^ ^134^ ^]^ Copyright 2024, John Wiley and Sons.

Activated macrophages often demonstrate the ability to change their characteristics, dividing into either pro‐inflammatory (M1) or anti‐inflammatory (M2) states, depending on the inflammatory microenvironment. Therefore, modulating macrophage polarization toward the M2 type while reducing the proportion of the M1 type population is an effective strategy for treating AKI. Curcumin is known to significantly downregulate the level of macrophage Mincle after AKI, promoting the transition of the macrophage phenotype from M1 to M2, and intervening in AKI by blocking the triggering of Mincle downstream factors spleen tyrosine kinase and nuclear factor κB.^[^ ^129^ ^]^ Nevertheless, the limited solubility and permeability of curcumin severely restrict its potential use in the therapy of AKI. Nanocarriers could enhance the utilization and effectiveness of curcumin. Indeed, researchers have developed various nanocarriers encapsulating curcumin to enhance its renal targeting ability. For instance, leveraging the intrinsic properties of hyaluronic acid targeting CD44 receptors, Hu et al.^[^ ^130^ ^]^ proposed a hyaluronic acid targeting CD44 receptors, developing an epithelial cell‐targeted polymer prodrug (HA‐curcumin, HA‐cur), which enhanced the concentration of curcumin in the kidney by a factor of 13.9 compared to free curcumin. This represents a new and efficient method of administering drugs applicable to HA‐based polymer prodrugs with significant potential for treating kidney diseases. In addition, in response to the overexpression of matrix metalloproteinase‐2 (MMP‐2) in the kidney during AKI, researchers crafted an MMP‐2 enzyme‐triggered polymer prodrug. Employing the MMP‐2 reactive peptide PVGLIG enabled the polymer prodrug to swiftly release curcumin after arriving at the target tissue, enhancing the drug levels.^[^ ^131^ ^]^ This enzyme‐triggered drug release strategy, while increasing the drug concentration in lesion tissues, can also reduce systemic toxicity, which is important for the treatment of AKI. Lipophilic cationic triphenylphosphine (TPP) serves as an effective mitochondrial targeting molecule, accumulating within mitochondria due to the Δφ (positive outside, negative inside) gradient. Currently, various TPP‐modified nanosheets have been utilized to administer different antioxidants. Triphenyl phosphine‐low molecular weight chitosan‐curcumin (TPP‐LMWC‐CUR; TLC) is one such nanosheet used to treat sepsis‐induced AKI. TLC has high water solubility and a relatively low molecular mass, which quickly distributes in the kidney tissue where it is specifically internalized by renal TECs through its interaction with the Megalin receptor and LMWC. Because of the strong buffering value of LMWC and the delocalized positive charge of TPP, intracellular TLC further transports curcumin to mitochondria.^[^ ^132^ ^]^ Polydopamine (PDA) is formed by the oxidation and self‐polymerization of the biomolecule dopamine under alkaline conditions. PDA is commonly utilized in biomedical research because of its strong chelating ability with metal ions and high biocompatibility. Zheng et al.^[^ ^133^ ^]^ proposed an ingenious method for synthesizing polydopamine‐coated manganese ferrite nanoparticles (PDA@MF NPs) as an antioxidant and anti‐inflammatory treatment structure (**Figure** **5**A). It was demonstrated that PDA@MF NPs successfully reprogrammed M1 macrophages into M2 macrophages (Figure 5B,C), synergistically scavenging ROS and producing O_2_. Xu et al.^[^ ^134^ ^]^reported that bilobetin (Bil) functionalized ultrasmall Cu_2−x_Se nanoparticles (CSPB NPs) can stimulate macrophages to express IL‐33, thereby alleviating the overactive inflammatory response caused by renal IRI (Figure 5D). Bil, a biflavonoid compound, can bind to the copper ions present on the surface of Cu_2‐x_Se nanoparticles (CSP NPs) to improve their water solubility and bioavailability. CSPB NPs demonstrate exceptional biocompatibility and efficiently enhance the expression of IL‐33, which facilitates the conversion of M1‐type macrophages into anti‐inflammatory M2‐type macrophages (Figure 5E–G), thereby reducing the secretion of pro‐inflammatory substances. CSPB NPs also have the ability to quickly gather at the damaged kidney, effectively reducing IRI. Furthermore, Shen et al. found that exosomes derived from mesenchymal stem cells (MSCs) (small membrane particles secreted by cells in a structured or induced manner, ranging from 40 to 1000 nm) have a protective effect on the kidneys in ischemia/reperfusion‐induced kidney injury. MSC‐derived exosomes (MSC‐Exos) are rich in proteins, including C‐C motif chemokine receptor‐2 (CCR2), which has a strong binding capacity with its ligand CCL2, acting as an intercellular messenger. CCR2 is typically expressed on monocytes and macrophages, where it promotes their migration and activation in the presence of extracellular CCL2. This study demonstrated that MSC‐Exos with high CCR2 expression reduced the concentration of free CCL2, inhibiting its ability to recruit or activate macrophages, which was largely dependent on the high expression of CCR2 protein.^[^ ^135^ ^]^

#### 2.4.5 Supplementation with Anti‐Inflammatory Factors

**Figure 6:**
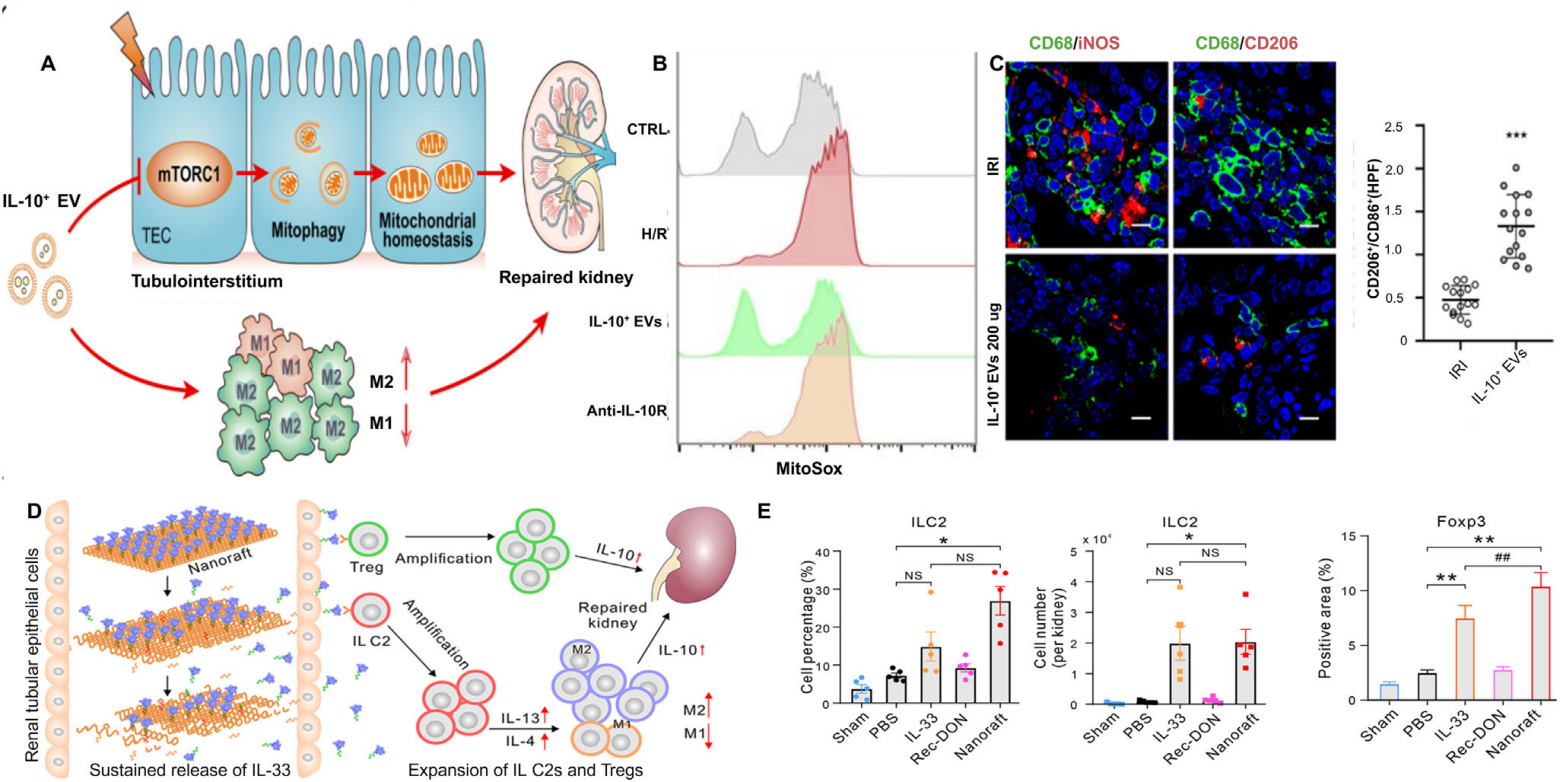
A) Schematic diagram of the preparation of IL‐10+ EVs and the therapeutic strategy of AKI. B) The production of ROS by mitochondria in TECs labeled with MitoSOX was studied using flow cytometry. C) Confocal images of CD68+ CD86+ or CD68+ CD206+ macrophages in kidney sections. The change in macrophages is shown by CD206+/CD86+. Reproduced with permission^[^ ^101^ ^]^ Copyright 2020, The American Association for the Advancement of Science. D) Nanorafts selectively gathered in the kidneys after an intravenous injection, and IL‐33 was released over time to cause rapid expansion of Tregs and ILC2s. E) Amount and percentage of ILC2 found in the kidneys of mice administered Sham‐, PBS‐, Rec‐DON‐, IL‐33‐, or nanoraft. Quantification of Foxp3‐positive Treg cells detected by IHC. Reproduced with permission.^[^ ^141^ ^]^ Copyright 2021, American Chemical Society.

The working principle of the fifth class of IMN is to balance the inflammatory imbalance in the kidneys by supplementing with cytokines. Cytokine immunotherapy has shown good effects for treating kidney diseases. For example, IL‐10, a potent immunomodulator, is endowed with significant anti‐inflammatory and tissue regeneration properties. Studies indicate that IL‐10 can safeguard against kidney damage caused by ischemia, cisplatin, or ureteral obstruction by curbing the generation of inflammatory cytokines and the penetration of immune cells,^[^ ^136^, ^137^ ^]^ suggesting that IL‐10 is a potential therapeutic approach to overcome present clinical difficulties in AKI management. Nevertheless, cytokines are prone to chemical and physical instabilities, unavoidably activating circulating leukocytes, potentially resulting in patient harm or reduced therapeutic effects.^[^ ^138^ ^]^ Addressing this challenge hinges on enhancing the durability of IL‐10 and selectively concentrating on the damaged kidney. Tang et al.^[^ ^101^ ^]^ synthesized extracellular vesicles (EVs) loaded with IL‐1, originated from macrophages, which can improve the durability of IL‐10 and efficiently target damaged kidneys (**Figure** **6**A), primarily because of the integrin and adhesion components on the surface of EVs. Research has shown that IL‐10+EVs not only target renal TECs (Figure 6B) but also macrophages in the renal tubulointerstitial space (Figure 6C), significantly alleviating renal IRI and hindering the progression from AKI to CKD. However, the levels of EVs are highly constrained by the extent of the injury. Thus, strategies that enhance the ability of EVs to traverse the glomerular filtration barrier as much as possible could prove highly beneficial. Specifically, IL‐10+EVS promotes mitosis by inhibiting the activity of the mammalian target of rapamycin (MTOR), thus maintaining mitochondrial stability in TECs. Furthermore, IL‐33, a member of the IL‐1 cytokine superfamily, plays a significant immunoregulatory role by activating multiple immune cells expressing the specific receptor ST2, and protects against renal IRI by promoting the expansion of ILC2s and Tregs.^[^ ^139^, ^140^ ^]^ However, one of the principal challenges in using IL‐33 for treating ischemic AKI is the specific delivery of cytokines to the kidneys and continuously stimulating the swift proliferation of ILC2s and Tregs. The emergence of DNA origami nanostructures (DONs) has enabled the preferential uptake of IL‐33 by the kidneys, making it a suitable platform for kidney‐targeted delivery. Specifically, researchers constructed a cytokine delivery platform based on a DNA nanoframework by meticulously arranging IL‐33 nanoarrays on rectangular DNA origami, achieving targeted delivery of IL‐33 specifically to the kidneys (Figure 6D). IL‐33 from Nanoraft's prolonged retention and sustained release in the kidneys led to the rapid proliferation of ILC2s and Tregs (Figure 6E), demonstrating its effectiveness in treating ischemic AKI.^[^ ^141^ ^]^ Nanorafts demonstrate superior therapeutic efficacy for ischemic AKI compared to free IL‐33 treatment, even with reduced treatment intensity.

#### 2.4.6 Regulating Oxidative Stress

**Figure 7:**
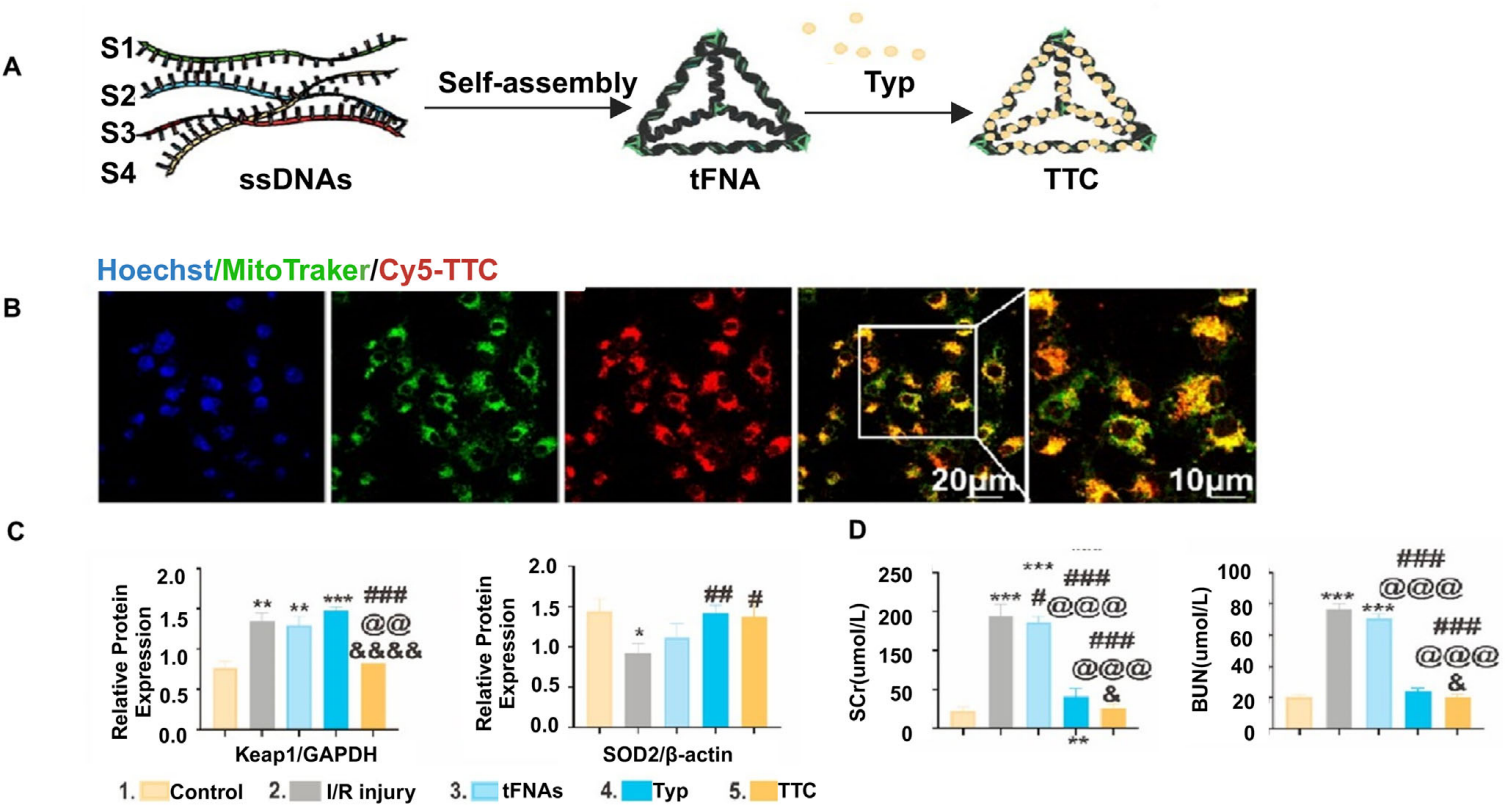
A) Diagram illustrating the process of producing tFNAs and TTCs. B) The coexistence of mitochondria and the TTC was detected through confocal microscopy. Red, TTC; blue, nuclei; green, cytoskeleton. C) Quantification of associated proteins in five groups: the Sham group, AKI+saline group, AKI+tFNAs group, AKI+Typ group, and the AKI+TTC group. TTC treatment decreased mitochondrial oxidative stress by activating the Keap1/SOD2 signaling pathway and attenuated fibrotic progression following IRI. D) Measurement of serum BUN and Scr levels in mice belonging to five groups. Reproduced with permission.^[^ ^143^ ^]^ Copyright 2023, American Chemical Society.

Oxidative stress (ROS generation) is another important driver in the development of AKI and plays a critical function in controlling immune and intrinsic cells. Increased ROS production leads to increased secretion of proinflammatory cytokines, enhanced polarization of M1 macrophages, and increased infiltration of inflammatory cells in the kidney.^[^ ^142^ ^]^ Researchers have designed various engineered nanosystems to eliminate excess ROS, ultimately mitigating inflammation. Nanoparticles developed on the basis of antioxidative enzymes and ROS scavengers have shown good results in clearing excess ROS and protecting cells from hydrogen peroxide‐induced oxidative damage. Concurrently, the use of generated ROS to prepare ROS‐responsive drug release systems has achieved satisfactory results in targeting organs damaged by AKI. In addition to neutralizing excess ROS, the preparation of nanocarriers encapsulating Nrf2 activators to reduce ROS production has received widespread attention. For example, DNA, with its rich nucleophilic groups and high electrophilic activity, can function as a strong ROS scavenger. However, DNA is limited by its diminished stability and swift deterioration due to the widespread presence of nucleases in the body. Yan et al.^[^ ^143^ ^]^ developed tetrahedral framework nucleic acid (tFNA) as a vehicle and combines typhaneoside (Typ) to develop the tFNA‐Typ complex (TTC) for antioxidant therapy in AKI (**Figure** **7**A). The TTC was found to have the ability of endocytosis and target mitochondria(Figure 7B) to repair mitochondria morphology function (Figure 7C). The kidney injury was mitigated and renal function was recovered after IRI‐AKI (Figure 7D). Furthermore, many transition metal compounds can clear ROS by receiving electrons from them. Presently, the primary types of transition metal nanocomplexes employed for AKI therapy are molybdenum (Mo)‐based polyoxometalate (POMs) and titanium‐MXenes.^[^ ^144^, ^145^ ^]^ For example, multivalent state‐based Mo compounds (mainly including Mo(III), Mo(IV), Mo(V), and Mo(VI)) have great potential for ROS removal.^[^ ^146^ ^]^ Recently, Ni et al.^[^ ^144^ ^]^ created a Mo‐based POM for treating AKI. With an ultra‐small hydrodynamic diameter (<10 nm) based on nanoclusters, the POM accumulated in the kidneys and efficiently decrease oxidative damage to DNA and lipid peroxidation, thereby leading to a successful recovery of renal function. Recently, Zhao et al.^[^ ^145^ ^]^ used ultrathin Ti3C2 MXene nanosheets (TPN) for the antioxidant therapy of AKI by effectively removing ROS. Compared to sacrificial agents for ROS, antioxidant enzymes (such as SOD, CAT) are more effective and durable because they catalyze ROS without being consumed by them. uang and his team have for the first time used ultra‐small tungsten‐based nanodots (TWNDs) to passively target mitochondrial therapy for AKI by protecting mitochondria and increasing mitochondrial autophagy to maintain mitochondrial function. Moreover, TWND can significantly diminish the apoptosis of renal tubule cells and the infiltration of macrophages.^[^ ^147^ ^]^ Currently, various antioxidant nanoenzymes have been used for the treatment of AKI, mainly including cerium oxide enzymes and noble metal enzymes. Nanoenzymes generally exhibit antioxidant enzyme activity and have the ability to eliminate other ROS through sacrificial or alternative mechanisms. Fortunately, ultra‐small‐sized gold,^[^ ^7^ ^]^ platinum,^[^ ^8^ ^]^ and other nanoparticles have been devised for treating AKI. Due to their antioxidant enzyme properties, these nanoparticles frequently passively approach renal tissues to remove various ROS. Among them, platinum nanoparticles are notably focused upon for their wide‐spectrum and effective antioxidant actions, imitating various enzymes such as catalase, superoxide dismutase, and NADH coenzyme Q reductase.^[^ ^148^ ^]^ Platinum has the high stability of noble metals and is catalytically active across a wide range of pH and temperatures.^[^ ^149^ ^]^ In addition, ROS‐responsive drug release achieves precise targeting of ROS. The SS‐31 peptide exerts an antioxidant impact on organ dysfunction caused by sepsis‐induced AKI via preserving mitochondrial homeostasis and suppressing cell apoptosis.^[^ ^150^ ^]^ However, SS‐31 has a brief half‐life, limited bioavailability, and inadequate specific biodistribution. Liu and others^[^ ^151^ ^]^ utilized ROS‐cleavable thioketal bonds to conjugate SS31 with L‐serine‐modified chitosan (SC), developing a precise stepwise targeting prodrug. SC‐TK‐SS31 is designed for effective AKI treatment, where chitosan, L‐serine, and SS31 endow the carrier with targeting capabilities toward AKI kidneys, renal tubular cells, and mitochondria. Specifically, chitosan with a positive charge can attach to heparin sulphate and sulfated glycosaminoglycans that have a negative charge in the fenestrae and glomerular basement membrane. Due to its renal targeting ability, SC is highly taken‐up and retained in the damaged kidneys of AKI mice. L‐serine in SC reaches the renal tubules and binds to kidney injury molecule‐1, causing SC‐TK‐SS31 to accumulate, persist, and internalize in damaged renal TECs. Upon damage to renal TECs, SS31 is released into the cytoplasm, co‐localizing at mitochondria to reduce oxidative stress and apoptosis in kidney TECs. The Nrf2 pathway is essential for controlling oxidative stress and the Nrf2‐mediated redox pathway has been a hot topic of research in recent years. Nrf2, as a transcription factor that responds to stress, efficiently stimulates the expression of genes, reducing ROS generation. OltiPraz is a potent Nrf2 activator, but its activating effect when used alone is weak. Yu et al.^[^ ^152^ ^]^ used the high accumulation of PLGA nanoparticles in ischemia‐reperfusion kidneys to design PLGA nanoparticles encapsulating OltiPraz. PLGA‐OltiPraz nanoparticles significantly enhanced the expression of Nrf2 and its downstream proteins, suggesting that PLGA‐OltiPraz nanoparticles can activate the Nrf2 signaling pathway and target renal tubules. PLGA‐Oltipraz NPs serve as a passive targeting strategy for the damaged kidney following IRI, characterized by precise particle size selection. Nonetheless, the evaluation period for its biocompatibility and safety is still relatively brief.

#### 2.4.7 Modulating the Tolerogenic Immune Response

**Figure 8:**
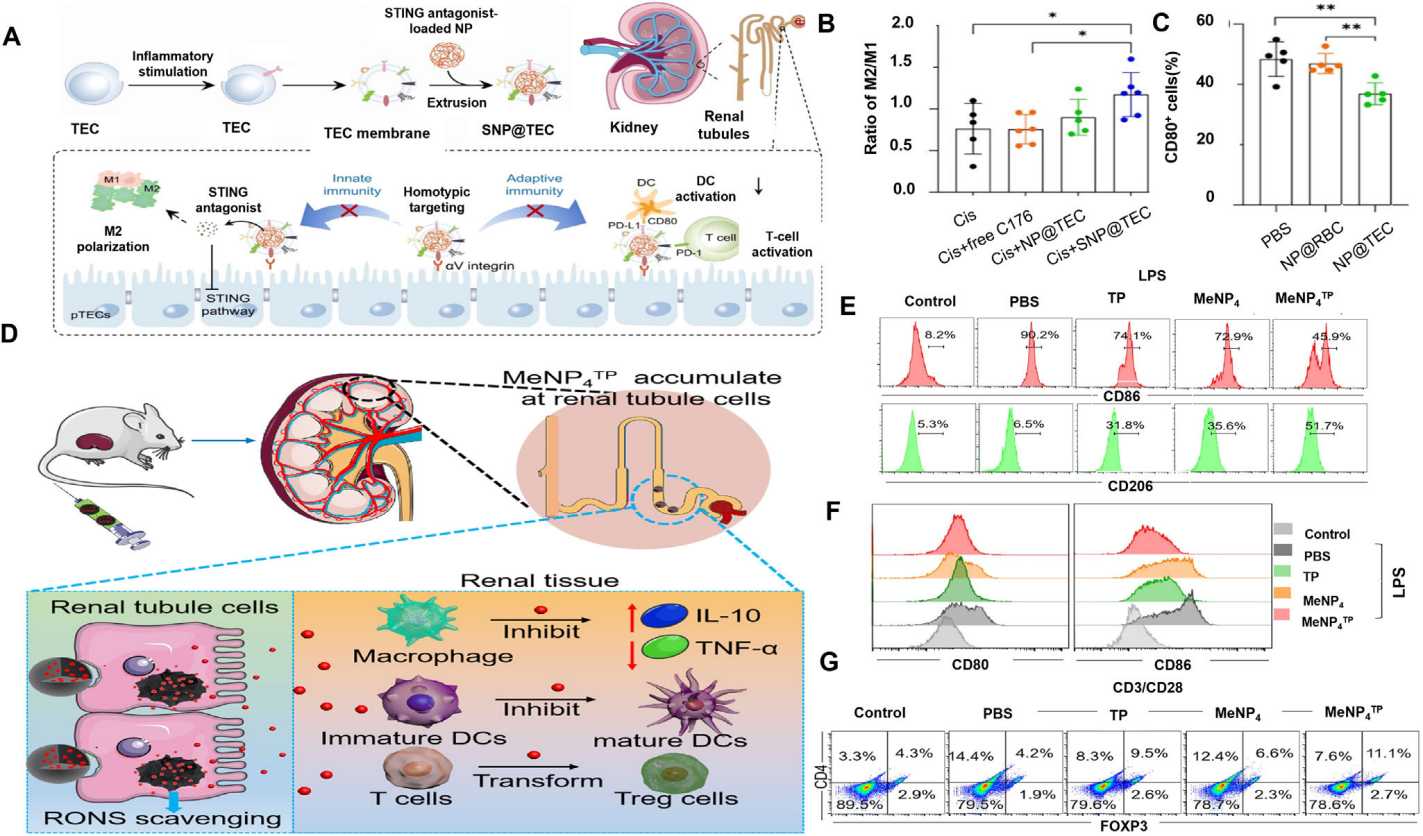
A) Schematic diagram of SNP@TECs used for orchestrating innate and adaptive immunity for inflammation suppression. B) Flow cytometric analysis of the ratio of M2‐like macrophages (CD206+ cells) to M1‐like (CD86+ cells) macrophages in the kidneys. C) Percentage of CD80+ BMDCs after different treatment in the presence of LPS for 1 h. Reproduced with permission.^[^ ^100^ ^]^ Copyright 2024, Elsevier. D) The effectiveness of MeNP4^TP^ in treating AKI. E) LPS resulted in the conversion of a significant majority of RAW 264.7 cells into the M1 phenotype in the PBS group, which exhibited greater levels of CD86 compared to the control group. The levels of CD86 were reduced and the generation of CD206 was elevated in RAW 264.7 cells following therapy with TP, MeNP4, or MeNP4^TP^. F) Mature DCs isolated from the bone marrow cells of normal C57BL/6J mice were cultured with LPS for 24 h to stimulate their maturation. Subsequently, the cells were cultured with TP, MeNP4, or MeNP4^TP^ for an additional 24 h. The administration of LPS resulted in an augmentation of the CD80 and CD86 quantities in DCs. Following the administration of TP, MeNP4, or MeNP4^TP^, the levels of CD80 and CD86 were significantly lowered. Notably, treatment with MeNP4^TP^ resulted in the most substantial decrease in CD80 and CD86 levels. G) Flow cytometry analysis of the percentage of Treg cells. Reproduced with permission.^[^ ^156^ ^]^ Copyright 2024, Elsevier.

Because of the complex inflammation regulatory network formed by diverse immune cells in the kidney, simply manipulating the activity of one type of immune cell is inadequate for treating kidney injury. Therefore, developing combined therapeutic strategies that simultaneously scavenge ROS and regulate the functions of multiple immune cells will be an effective approach for treating AKI. Promoting the differentiation of T cells into anti‐inflammatory T cells and enhancing the tolerance of DCs to augment, regulate, or suppress the corresponding immune responses are also noteworthy. Within the inflammatory setting, proximal TECs induce a significant increase in the level of programmed death ligand 1 (PD‐L1), which competes with CD80 on DCs, hindering T cell expansion by obstructing co‐stimulatory signals.^[^ ^153^ ^]^ Stimulated proximal TECs expressing PD‐L1 can also prompt the transformation of CD4+ T cells into an anti‐inflammatory phenotype and diminish the activity of CD8+ T cells.^[^ ^154^, ^155^ ^]^ Therefore, Shen et al.^[^ ^100^ ^]^ designed and fabricated a nanocarrier wrapped in a TEC membrane loaded with a STING inhibitor (SNP@TEC), which could alleviate AKI by simultaneously inhibiting innate and adaptive inflammation (**Figure** **8**A). Initially, due to the αV integrin on the TEC membrane surface, SNP@TEC enhanced accumulation in the renal tubules through homing targeting after systemic administration. Upon reaching the kidney, SNP@TEC mitigated the innate immune response by relieving the STING‐induced inflammatory response with its carried inhibitor and directing macrophages toward an anti‐inflammatory M2 phenotype (Figure 8B). Moreover, because of the presence of PD‐L1 on the TEC membrane surface, SNP@TEC could also inhibit T cell activation and induce T cells to differentiate into regulatory T cells by blocking CD80 on DCs (Figure 8C), thereby initiating an anti‐inflammatory adaptive immune reaction. In two AKI mouse models induced by cisplatin and ischemia/reperfusion, intravenous injection of SNP@TECs considerably mitigated renal injury and restored kidney function. While the current design demonstrates effective treatment for AKI, this biomimetic nanoplatform could further enhance the therapeutic performance by enhancing targeting capability via fusion with inflammatory cell membranes and increasing drug loading efficiency. Moreover, triptolide (TP), functioning as an anti‐inflammatory medication, hinders the development and activity of immune cells, including macrophages, T cells, and DCs. Polydopamine nanoparticles have the potential to act as carriers for TP by forming hydrogen bonds between the C‐14 hydroxyl group of TP and the ortho‐hydroxyl group of polydopamine. This interaction can effectively hinder the activity of pro‐inflammatory immune cells. MeNP4^TP^ was synthesized through the incorporation of TP into MeNP4 by Han and colleagues (Figure 8D).^[^ ^156^ ^]^ When MeNP4^TP^ is intravenously injected into mice with cisplatin‐induced AKI, it selectively accumulates in renal tubular cells based on its size specificity. MeNP_4_^TP^ effectively scavenges ROS because of the antioxidant properties of dopamine, inhibits the activity of macrophages (Figure 8E) and DCs (Figure 8F), and promotes the production of regulatory T cells via the discharge of TP (Figure 8G). Therefore, MeNP_4_^TP^ has a significant therapeutic effect on AKI and has great potential for clinical application. Moreover, the glomerular filtration barrier represents a major obstacle to the application of MeNP_4_^TP^ therapy. Under kidney injury conditions, the damage to the barrier, especially the widened endothelial gaps, may promote MeNP_4_^TP^ accumulation in renal cells and structures.

## 3 Conclusions and Perspectives

At present, no established treatment exists for AKI or for halting its progression to chronic kidney disease (CKD).^[^ ^157^ ^]^ Clinical treatment currently relies on supportive care and renal replacement therapy (RRT).^[^ ^158^ ^]^ Specific strategies involve maintaining adequate renal blood flow through hemodynamic monitoring, ensuring proper nutritional support (sufficient protein intake), and optimizing blood glucose control. Monitoring nephrotoxic drug use closely and discontinuing such medications to avoid aggravating AKI. In cases of life‐threatening water, electrolyte, or acid‐base imbalances, RRT must be implemented to reduce the mortality associated with AKI.^[^ ^147^ ^]^ Over the past few decades, immunotherapy based on nanomaterials has experienced rapid development and has shown considerable potential. IMNs that regulate the immune microenvironment of AKI are becoming increasingly popular. Nanosystems constructed from multifunctional composite materials can accurately and efficiently modulate the local immune microenvironment of AKI, thereby facilitating its repair. As discussed in this review, new evidence emphasizes the good results achieved in AKI treatment through the synergistic effects of reducing pro‐inflammatory cytokine production, neutralizing excessive cytokines, modulating immune cell infiltration, adjusting macrophage polarization and activity, supplementing anti‐inflammatory cytokines, regulating oxidative stress, and modulating tolerogenic immune response. Despite the great potential of IMNs, several challenges must be addressed before their clinical translation.

(1) A systematic study of the safety of nanosystems is a top priority. During the creation of innovative nanosystems, ensuring application safety through design and preparation is paramount. When dealing with this situation, it is crucial to prioritize improving the accuracy of targeting, optimizing the compatibility with living organisms, and minimizing the effects on both the intended target and unintended tissues. It is crucial to guarantee that both patients and medical professionals derive advantages from this groundbreaking technology.

(2) Given that renal parenchymal cells, especially renal TECs, exhibit several characteristics of immune cells and express components that are crucial for innate and adaptive immune cascades in kidney inflammatory responses, current IMNs for treating AKI primarily focus on targeting interactions between renal TECs and immune cells in the kidney. The scarcity of nanoresearch directly targeting renal immune cells may be largely due to the unique glomerular filtration barrier of the kidney and our insufficient comprehension of the proportions of various immune infiltrating cells in AKI at various stages and causes. Nevertheless, the rapid progress of single‐cell RNA sequencing (scRNA‐Seq) technology has enabled the detailed characterization of heterogeneous cell populations.^[^ ^159^ ^]^ Thus far, scRNA‐Seq has offered valuable understanding of the complexity of the nephritic renal parenchyma and immune cells in IgA nephropathy, diabetic nephropathy, lupus nephritis, and ANCA‐associated glomerulonephritis. Single‐strand RNA sequencing research has proved the existence of resident memory B cells and CD4+ T cells in renal biopsy samples obtained from patients with IgA nephropathy, diabetic nephropathy, and lupus nephritis, indicating a common mechanism of renal inflammation development across different etiologies of kidney diseases. The proportions of various immune‐infiltrating cells may vary with the disease and its stage, leading to heterogeneity in the inflammatory reaction of patients with kidney disease. Future scRNA‐Seq studies should focus on identifying therapeutic targets shared among clinical entities or specific targets for different AKI causes and stages, aiming for precise medical treatment methods.^[^ ^160^ ^]^

(3) It was initially believed that the absorption of nanoparticles by phagocytic cells in the mononuclear phagocyte system should be avoided to increase drug accumulation at the lesion site. However, it has recently been shown that phagocytic cell uptake of nanoparticles is beneficial for nanoparticle targeting of immune cells such as macrophages and DCs. Therefore, for different applications, the characteristics of nanoparticles, such as size, surface chemistry, and surface charge, can be adjusted to mediate homing or to avoid phagocytosis by phagocytic cells. However, delivering nanomedicine to specific immune cell subpopulations poses a significant challenge.

(4) Accurate assessment of the therapeutic efficacy of IMNs for treating AKI is another significant challenge currently faced. In situ longitudinal monitoring of molecular events in the kidney is difficult with static analysis‐based in vitro diagnostic methods. The progression of kidney injury is closely linked to redox imbalance and oxidative stress. Dynamic monitoring of the kidney's redox state will provide valuable information for diagnosing renal dysfunction and evaluating therapeutic efficacy. Molecular imaging is a noninvasive technique for real‐time identification of disease occurrence and progression.^[^ ^161^, ^162^, ^163^ ^]^ Over the past few years, various probes have been created to identify ROS, effectively addressing the limitations of conventional techniques. The techniques are primarily categorized as near‐infrared fluorescence imaging, chemiluminescence imaging, and photoacoustic imaging, all of which provide high spatial‐temporal resolution and extremely high sensitivity^[^ ^164^, ^165^, ^166^ ^]^ and can measure subtle changes in the concentration of biomarkers at disease sites through molecular probes.^[^ ^167^, ^168^ ^]^ Compared to traditional methods, these techniques offer advantages such as prompt diagnosis, great imaging depth, efficient renal clearance rate, excellent biosafety, minimal background noise, and immediate detection. For example, Pu et al. created a collection of near‐infrared optical imaging probes (MRPs1‐3), with the imaging results based on ·O_2_^−^ being the best.^[^ ^169^ ^]^

(5) In this article, we summarize the use of nanomaterials in immunomodulation‐based therapies for AKI. Additionally, kidney diseases are highly diverse, and immune factors play a role in the pathogenesis of most kidney diseases. Therefore, immunomodulatory therapy is not limited to AKI, and the combination of nanoparticles with immunotherapy could be considered to enhance the treatment of other kidney diseases. Additionally, renal nanomedicine, with its distinctive properties and versatility, offers a promising approach to counter the progression of AKI to CKD. Nanomaterials have shown significant potential to alleviate pathways contributing to CKD, including excessive ROS production, ferroptosis, EV‐mediated signaling, and p53‐mediated cellular processes. Nanomedicines can specifically target the kidney, cells, and organelles according to the pathological environment, minimizing side effects and maximizing therapeutic benefits. Nanotechnology not only offers drug delivery support but also substantial promise for early diagnosis and monitoring of kidney diseases at different stages of development. Research has revealed that surface‐engineered nanoparticles can amplify fluorescence or magnetic signals, allowing clinicians to accurately diagnose diseases in their early stages, especially for monitoring AKI.^[^ ^170^ ^]^ Beyond drug delivery and imaging diagnostics, nanotechnology integrated with gene therapy provides a promising new pathway to cure kidney diseases by reversing renal fibrosis and repairing genetic mutations. One study demonstrated the use of lipid nanoparticles for delivering siRNA, effectively inhibiting the fibrosis‐related TGF‐β1 gene in diabetic nephropathy.^[^ ^171^ ^]^ The results indicated a 40% reduction in kidney tissue fibrosis and remarkable protection of renal function. Moreover, nanoparticles facilitate the delivery of CRISPR‐Cas9 systems, enabling effective repair of genetic mutations linked to kidney diseases.^[^ ^172^ ^]^ Future advancements are anticipated to integrate therapeutic and diagnostic capabilities, leading to multifunctional nanoparticle platforms that efficiently target kidney injury sites and monitor treatment efficacy in real time, offering more comprehensive data for clinical applications.

The clinical application of nanomedicines in AKI still faces significant challenges, such as species differences between model animals and humans, optimization of nanomaterial properties, limited exposure of nanomedicines to target cells or tissues, as well as concerns regarding biocompatibility and safety. In related experimental studies, scientists typically conduct extensive experiments and characterizations to obtain data on nanomaterials and continuously optimize parameters to achieve the optimal state of the materials. Artificial intelligence (AI) algorithms can efficiently process large datasets, rapidly perform calculations and analyses, and make intelligent decisions. With the rapid development of AI, this technology has been widely applied in the field of materials science. The use of AI tools not only enhances the efficiency of nanomaterial design and reduces trial‐and‐error costs, but also helps researchers determine the most appropriate synthesis methods and extract deeper information from material characterization data.^[^ ^173^ ^]^ Through systematic screening and reliable synthesis methods, various biophysical and chemical properties of nanoparticles can be addressed, enabling precise control of nanoparticle production homogeneity and achieving large‐scale and high‐throughput production. Additionally, the protein corona has an important influence on the biodistribution, cellular uptake, stability, and efficacy of nanomedicines, necessitating further research into its formation and regulatory mechanisms to enhance the performance of nanomedicines. Moreover, large animal models that mimic the heterogeneity and anatomical features of human kidney diseases should be employed to effectively evaluate their efficacy. Stringent standards should also be used to assess the fundamental parameters of nanomedicines, improving the safety and efficacy of nanomaterials. Finally, more nephrology experts should be invited to participate in research to increase their collaboration and interdisciplinary contributions, thereby better facilitating clinical translation and laying the foundation for precise treatment of AKI. Overall, as research on IMNs for treating AKI transitions from basic scientific study to clinical application, addressing the remaining challenges is crucial.

## Conflict of Interest

The authors declare no conflict of interest.
